# Dualsteric Agonist for M2 Muscarinic Receptor Causes Oxidative Stress and Mitochondrial Alteration in Human Glioblastoma Cancer Stem Cells

**DOI:** 10.1111/jnc.70454

**Published:** 2026-05-16

**Authors:** Claudia Guerriero, Chiara De Nuccio, Maria Petrone, Teresa Rinaldi, Angela Cirigliano, Sergio Visentin, Antonietta Bernardo, Luciano Conti, Carlo Matera, Marco De Amici, Clelia Dallanoce, Ada Maria Tata

**Affiliations:** ^1^ Department of Biology and Biotechnologies Charles Darwin Sapienza University of Rome Rome Italy; ^2^ Research Coordination and Promotion Service Istituto Superiore di Sanità Rome Italy; ^3^ National Center for Research and Preclinical and Clinical Evaluation of Drugs Istituto Superiore di Sanità Rome Italy; ^4^ Department of Cellular, Computational and Integrative Biology – CIBIO University of Trento Trento Italy; ^5^ Department of Pharmaceutical Sciences University of Milan Milan Italy; ^6^ Research Center of Neurobiology “Daniel Bovet” Sapienza University of Rome Rome Italy

**Keywords:** cancer stem cells, cellular stress, Dualsteric agonist, glioblastoma, M2 muscarinic receptor, mitochondria

## Abstract

Glioblastoma (GB) is the most malignant form of human brain tumor, characterized by heterogeneous cell populations, including undifferentiated cells defined as GB stem cells (GSCs), responsible for initiating the neoplastic process as well as recurrence. Previous studies demonstrated that the activation of M2 muscarinic acetylcholine receptor (M2 mAChR) by the orthosteric agonist arecaidine propargyl ester (APE) and the dualsteric agonist Iper‐N‐naphthalimide (N‐8‐Iper) caused a significant decrease in cell proliferation and survival in both GSCs and GB cell lines. Interestingly, N‐8‐Iper activates M2 mAChR with higher efficacy and at a lower concentration than APE. The work aimed to better investigate the mechanisms downstream of M2 mAChR activation by both agonists responsible for cytotoxic and pro‐apoptotic effects in both U251 cell line and G166 cells (GSCs). To this end, we assessed mitochondrial function by using cell‐based assays. Our results demonstrate the ability of N‐8‐Iper, both at the high (100 μM) and low (25 μM) dose, to induce alteration of mitochondrial morphology and activity, affecting both cellular respiration and ROS production in U251 and G166 cells. Instead, APE causes the same alterations but only in the U251 cell line. Given the relevance of lipid metabolism analysis in the study of cancer, lipid droplets (LDs) were evaluated in the presence or absence of the two M2 agonists. LDs accumulation within the cells was detected after N‐8‐Iper treatment in both cell lines, whereas APE produced similar effects only in the U251 cell line. No cytotoxic effects and mitochondrial alteration were detected on normal human astrocytes. These results clearly suggest that N‐8‐Iper has a more potent action on GSCs than APE, thus making this dual‐acting agonist a promising muscarinic ligand able to better characterize the inhibitory effects of the M2 muscarinic receptor in glioblastoma cells as well as in other tumor types.

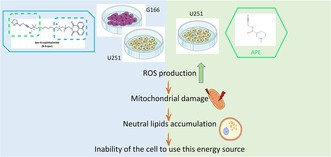

## Graphical abstract

Dualsteric agonist N8‐iper shows cytotoxic effects on glioblastoma cancer stem cells, but not on human normal astrocytes. Lipid accumulation appears as a consequence of cytotoxic stress; in fact, they are not present in healthy astrocytes.

**Abbreviations**
ACh: acetylcholine
APE: arecaidine propargyl ester
ATP: adenosine triphosphate
CHOL: cholesterol
CHOL‐E: cholesterol ester
CNS: central nervous system
CSCs: cancer stem cells
DAG: diacylglycerol
DCFDA: dichloro‐dihydro‐fluorescein diacetate
DIV: days in vitro
FAS: fatty acid synthase
GB: glioblastoma
GPCR: G protein coupled‐receptor
GSCs: glioblastoma cancer stem cells
LDs: lipid droplets
mAChRs: muscarinic acetylcholine receptors
MAGL: monoacylglycerol lipase
N‐8‐Iper (N8): Iper‐8‐naphthalimide
NAC: N‐acetyl‐L‐cysteine
OD: optical density
PVDF: polyvinylidene difluoride
ROS: reactive oxygen species
SD: standard deviation
SEM: standard error of the mean
TAGs: triacylglycerols
WHO: World Health Organization

## 1 Introduction

Glioblastoma (GB) is defined as grade IV astrocytoma (2016 World Health Organization Classification of Tumors of the Central Nervous System), and is the most common and aggressive type of brain tumor in humans (Louis et al. 2016). GB has a high ability to infiltrate throughout the brain parenchyma and to induce new angiogenesis in hypoxic regions, promoting tumor invasiveness (Rong et al. 2006). GB is also characterized by intra‐tumoral heterogeneity. That is due, at least in part, to the different microenvironments within the various tumor regions. The diverse oxygen pressure, blood vessel density, the presence of growth factors and the irradiation may contribute to the epigenetic differences observed in cancer cell populations. This finding, together with the genetic background of the patients, is among the possible key factors that may contribute to tumor growth, progression, and high resistance to therapy, typical of GB (Inda et al. 2014). GB is distinguished by a hierarchical cellular organization, and by a rare cell population defined as Glioblastoma stem cells (GSCs) (Lathia et al. 2015). GSCs represent the tumorigenic component of GB; they can self‐renew and generate progeny of multiple lineages, and are capable of initiating tumors and recapitulating tumor heterogeneity when transplanted in immune‐compromised mice (Gimple et al. 2019).

It is now accepted that acetylcholine (ACh) not only acts as the neurotransmitter of the neuronal cholinergic system, but is also a signaling molecule capable of modulating cell proliferation, survival, and migration in different cell types (Sastry 1978). In humans, ACh and choline acetyltransferase have been found in non‐neuronal tissues, as in immune system cells as well as in epithelial, mesothelial, and endothelial cells (Wessler and Kirkpatrick 2009). Interestingly, the expression of muscarinic acetylcholine receptors (mAChRs) is well documented in various metastatic and primary tumors (Calaf et al. 2022). In this respect, our research group has studied the effects associated with the activation of the M2 mAChR subtype in several tumor types such as GB, neuroblastoma, bladder and breast cancer, and ovarian carcinoma (Guerriero et al. 2021; Pacini et al. 2014; Lucianò et al. 2020; Taggi et al. 2022).

In general, M2 mAChRs are a subtype of acetylcholine receptors that play a key role in the parasympathetic regulation of the heart. Beyond the heart, M2 mAChRs also function in several other tissues where they mainly act as modulators rather than primary effectors. In the nervous system, M2 receptors are commonly located on presynaptic nerve terminals, where they act as autoreceptors that inhibit further release of acetylcholine. M2 receptors mainly serve inhibitory and regulatory roles in autonomic and smooth muscle function.

Activation of M2 mAChR, a typical inhibitory subtype of the family of muscarinic receptors, was found to be relevant in reducing GB cell proliferation and survival (Ferretti et al. 2012). M2 mAChR activation by the orthosteric agonist Arecaidine Propargyl Ester (APE) causes the arrest of cell cycle progression in a time‐ and dose‐dependent manner, both in GB stable cell lines (U87 and U251) and in GSCs (GB7 and GB8) (Ferretti et al. 2013; Alessandrini et al. 2015). Moreover, our results proved that APE induces intracellular reactive oxygen species (ROS) production in GB stable cell lines; it also causes chromosomal aberrations and DNA double‐strand breaks (Di Bari et al. 2015).

Since the above‐mentioned effects of M2 mAChR activation were achieved only at high concentration (100 μM) of the orthosteric agonist APE, and considering the pleiotropic roles of mAChRs in several tissues, we explored other muscarinic activators. Like the other G protein‐coupled receptors, mAChRs possess several extracellular allosteric binding sites (Gregory et al. 2007). At variance with the orthosteric site, the allosteric regions of the different receptor subtypes are characterized by higher heterogeneity in amino acid sequences and spatial conformation (Mohr et al. 2010). Besides positive allosteric modulators, new ligands called “dualsteric agonists” of mAChRs have been synthesized and tested in the last two decades, which bind simultaneously both the orthosteric and allosteric sites of the receptor protein. For a given dualsteric derivative, this recognition mode combines a high binding affinity (from the orthosteric site) with a higher degree of selectivity (from the allosteric site) for a specific receptor subtype (Kamal and Jockers 2009). Recently, we investigated synthetic M2 dualsteric agonist Iper‐8‐naphthalimide (N‐8‐Iper) (Guerriero et al. 2021; Cristofaro et al. 2018) that was found to affect GSCs survival, inducing cytotoxic effects and DNA damage in GSCs, in particular in the p53 mutated cell line (Cristofaro et al. 2018). Moreover, N‐8‐Iper counteracted drug resistance in two GSC lines, GB7 cells and the more resistant G166 cells (Guerriero et al. 2021).

Based on these results, in this work, we explored the downstream mechanisms of M2 mAChR activation by the two agonists APE and N‐8‐Iper, responsible for the cytotoxic and pro‐apoptotic effects both in GSCs G166 cells and in the U251 cell line. Oxidative stress, mitochondrial activity, and lipid accumulation were also investigated in both GB cell lines and normal human astrocytes, as controls. Our data show that M2 mAChR activation, particularly by N‐8‐Iper, impaired mitochondrial function and caused lipid accumulation in GB cells, but not in human astrocytes.

## 2 Materials and Methods

### 2.1 Cell Cultures

G166 and GB7 cells were obtained from human GBM biopsies (Rispoli et al. 2014; Conti et al. 2012; Pollard et al. 2009) and provided from Prof. Conti (CIBIO Trento, IT). The cells were cultured on laminin‐coated dishes (1 μg/mL; Sigma‐Aldrich, St. Louis, MO, USA) and maintained in serum‐free medium, consisting of DMEM/F12 (Sigma‐Aldrich, St. Louis, MO, USA) and Neurobasal medium (Gibco, Thermo Fisher Scientific, Waltham, MA, USA) (1:1, v:v) supplemented with 1% streptomycin, 50 IU/mL penicillin (Sigma‐Aldrich, St. Louis, MO, USA), 1% glutamine (Sigma‐Aldrich, St. Louis, MO, USA), 1% N2 supplement (Gibco, Thermo Fisher Scientific, Waltham, MA, USA), 2% B27 (Gibco, Thermo Fisher Scientific, Waltham, MA, USA), 20 ng/mL EGF (Recombinant Human Epidermal growth factor, Peprotec, London, UK), and 20 ng/mL FGF (Recombinant Human FGF‐basic, ABM, Richmond, Canada). The cell culture was maintained at 37°C in a 5% CO_2_ atmosphere and the experiments were performed within the 30 passages.

The human glioblastoma U251MG cell line is distributed as U373; cat. no. ECACC 08061901. They were cultured in DMEM (Sigma‐Aldrich, St. Louis, MO, USA) plus 10% fetal bovine serum (Sigma‐Aldrich, St. Louis, MO, USA), 50 μg/mL streptomycin, 50 IU/mL penicillin, 2 mM glutamine (Sigma‐Aldrich, St. Louis, MO, USA), and 1% non‐essential amino acids (Sigma‐Aldrich, St. Louis, MO, USA). The cells were maintained at 37°C in a 10% CO_2_ atmosphere and the experiments were perfomed within 30 passages.

The G166 cell line derives from a female patient and has been largely characterized (Pollard et al. 2009). It does not present IDH1/2 mutation. GB7 cells, reported in Supporting Information: figures, are p53 WT and MGMT mutated (Cristofaro et al. 2018). U251 cells are p53 mutated and present a PTEN deletion in heterozygous (Ferretti et al. 2012).

Human astrocytes, isolated from the human cerebral cortex (Sciencell Research Laboratories Carlsbad, CA, USA), were cultured on poly‐L‐lysine‐coated culture dishes (2 μg/cm^2^; Sciencell Research Laboratories Carlsbad, CA, USA) and maintained in Astrocyte Medium (Sciencell Research Laboratories Carlsbad, CA, USA) supplemented with 2% fetal bovine serum (Sciencell Research Laboratories Carlsbad, CA, USA), 1% astrocyte growth supplement (Sciencell Research Laboratories Carlsbad, CA, USA), and 1% penicillin/streptomycin solution (Sciencell Research Laboratories Carlsbad, CA, USA). The cultures were maintained at 37°C and 5% CO_2_ atmosphere.

### 2.2 Cell Treatments

Arecaidine Propargyl Ester (APE, Sigma‐Aldrich, Milan, Italy) is a synthetic compound obtained from arecaidine, a natural alkaloid derived from Areca nut. APE was used to selectively stimulate the M2 mAChR subtype. The ability of this agonist to bind the M2 mAChR subtype was previously demonstrated in GB stable cell lines (U87 and U251 cell lines) and in GSCs (GB7 and GB8 cells) by binding experiments and knockdown of the receptors by siRNA transfection pool (Ferretti et al. 2013; Alessandrini et al. 2015). Iper‐8‐naphthalimide (N‐8‐Iper) was synthesized according to a known literature procedure (Bock et al. 2012). Similarly to APE, the ability of N8‐Iper to selectively bind M2 mAChR subtype has been demonstrated by competition binding and M2 mAChR silencing experiments (Cristofaro et al. 2018). To compare the activity of two M2 receptor ligands, we first used both agonists at high concentration (100 μM), adding the lower dose of N‐8‐Iper (25 μM), producing significant effects in G166 cells, as previously assessed (Guerriero et al. 2021).

### 2.3 ROS Detection

ROS production was measured by 2,7‐dichlorodihydrofluorescein diacetate (DCFH‐DA; cat. no. ALX‐610‐022‐M050 Enzo Life Sciences, New York, USA) used as an oxidation substrate. DCFH‐DA is oxidized by peroxynitrite to the highly fluorescent product dichlorofluorescein (DCF). 2 × 10^4^ cells were plated in 96‐well plates, and next day, cells were incubated for 12 h with 100 μM APE, 100 μM N‐8‐Iper or 25 μM N‐8‐Iper. The ROS scavenger N‐acetyl‐L‐cysteine (NAC; Enzo Life Sciences) (10 μM, final concentration), added 2 h before the treatments, was used to inhibit ROS production. Then, the medium was removed, and cells were incubated with 10 μM DCFH‐DA for 30 min. The fluorescence was read at wavelengths of 485 nm of excitation and 530 nm of emission using the Glomax Multi Detection System (Promega Italia, Milan, Italy).

### 2.4 Mitochondrial Activity Assays

#### 2.4.1 Fluorescence Image Analysis (Mito ID Assay)

The cells (35 × 10^3^) were plated in μ‐Slide 8 wells (Ibidi, GmbH, Munich, Germany). The next day, the cells were treated with 100 μM APE, 100 μM N‐8‐Iper, or 25 μM N‐8‐Iper for 30 h. At the end of the treatment, cells were incubated with Hoechst 33342 (1:1000 in PBS, Thermo Fisher Scientific, Waltham, MA, USA) for 20 min, then were stained with Dual Detection Reagent (MITO ID Membrane Potential Detection Kit, cat. no. ENZ‐51018‐K100, Enzo Life Sciences, New York, USA), in accordance with the manufacturer's instructions. The cells were observed with the Zeiss Axio Imager Z1 Fluorescence Microscope with AxioVision 4.8 Digital Image Processing System (Zeiss, Wetzlar, Germany). The orange fluorescence emission associated with energized mitochondria with high mitochondrial membrane potential (MMP) can be detected using a rhodamine filter set (excitation = 540 nm, emission = 570 nm). The green fluorescence emission associated with depolarized mitochondria with low MMP can be detected using a fluorescein (FITC) filter set (excitation = 485 nm, emission = 530 nm). The images were acquired with the Axion Vision program (Zeiss, Wetzlar, Germany) using a 40× objective lens.

#### 2.4.2 Fluorescent Video Imaging Analysis

The cells (2 × 10^4^) were plated onto 25 mm glass coverslips. The next day, cells were treated with 100 μM APE, 100 μM N‐8‐Iper, or 25 μM N‐8‐Iper for 30 h. The mitochondrial membrane potential (MMP) and the mitochondrial morphology were analyzed by the video‐imaging technique using the fluorescent and potentiometric dye Tetramethylrhodamineethyl ester (TMRE) perchlorate (CAS115532‐52‐0, cat. no. ENZ52309, Enzo Life Sciences, New York, USA). TMRE was used at the final concentration of 30 nM. To reach dye saturation, cells were kept for 30 min in the presence of TMRE perchlorate before recording. The composition of the saline solution used for loading and recording was the following (mM): 140 NaCl, 5 KCl, 1 MgCl_2_, 2.5 CaCl_2_, 5.5 D‐glucose, 10 HEPES/NaOH at room temperature (RT), pH 7.4. An inverted microscope (Axiovert 135, Zeiss, Wetzlar, Germany) equipped with a 40× and 1.35 NA (Olympus, Tokyo, Japan) oil immersion objective was used. The appropriate excitation wavelength (535 nm) was applied by means of a monochromator (Polychrome II; Till Photonics, Munich, Germany), and the emission light (590 nm) was collected by a CCD, cooled digital camera (PCO; Sensicam, Kelheim, Germany), and recorded on the hard disk of a computer. The Imaging Workbench 6.0 software package (Indec BioSystems, Santa Clara, CA, USA) was used to record and analyze the data. The software allowed the measurement of the emission values along line profiles crossing mitochondria. A minimum of two peaks of amplitude was used to calculate the average amplitude of a given mitochondrion. Since the organelles were densely packed around a mitochondria‐free area corresponding to the nucleus and single mitochondria were detectable only in the periphery of a cell, for the evaluation of MMP, only filamentous mitochondria in this latter area were chosen for analysis. TMRE‐loaded cultures were also used to evaluate mitochondrial morphology and to classify the cells accordingly. Cell types considered for this analysis were cells with filamentous mitochondria, cells with filamentous and point‐like mitochondria, cells with filamentous and swollen mitochondria, cells with point‐like mitochondria, cells with swollen mitochondria.

### 2.5 Measurement of Mitochondrial Respiration

The Seahorse XFp analyzer (cat. no. AG‐103022‐100; Agilent Technologies, Wilmington, DE, USA) was used to study key cellular functions such as mitochondrial respiration and glycolysis through real‐time measurement of oxygen consumption rate (OCR) and extracellular acidification rate (ECAR) in cell cultures. Cells were plated in a Seahorse XFp cell culture microplate (U251 cell line: 5000 cells/well; G166 cells: 10000 cells/well) with a culture medium composed of DMEM, supplemented with 1 mM pyruvate, 2 mM glutamine and 10 mM glucose. The next day, cells were treated with 100 μM APE, 100 μM N‐8‐Iper or 25 μM N‐8‐Iper for 30 h. The kit used (cat. no. AG‐103010‐100; Cell Mito Stress Test Kit, Agilent Technologies, Wilmington, DE, USA) allowed to measure certain key parameters of mitochondrial function by sequentially adding, during the experiment, some modulators of mitochondrial respiration. The added modulators were 1 μM oligomycin (OLIG), 2 μM carbonyl cyanide‐4 (trifluoromethoxy) carbonyl cyanide 4‐(trifluoromethoxy) phenylhydrazone (FCCP), and 0.5 μM rotenone (ROT) + 0.5 μM antimycin A (ANT). Offline analysis by means of the Wave program (Agilent Technologies, Wilmington, DE, USA), of OCR obtained by using the Cell Mito Stress Test Kit allowed to measure multiple parameters, including basal respiration, ATP production and spare respiratory capacity.

### 2.6 Neutral Lipid Analysis by Oil Red O Staining

The fat‐soluble dye Oil Red O (cat. no. HY‐DY1096; DBA, Milan, IT) was used to detect lipid droplets (LDs). It has the appearance of a red powder soluble in isopropanol, which dissolves lipid molecules, turning them orange red in color. Cells were seeded on 12 mm coverslips onto 24 well‐plates at a density of 1 × 10^4^ cells/well. The next day, cells were treated with 100 μM APE, 100 μM N‐8‐Iper, or 25 μM N‐8‐Iper for 72 h. At the end of the treatment, the cells were fixed with 4% paraformaldehyde for 20 min at RT and washed three times in PBS. Then, cells were incubated with the working solution (Oil Red O: dH_2_O, 2:3, v:v) for 30 min and washed 5 times in PBS. Finally, cells were incubated with Hoechst 33342 (1:1000 in PBS, H3570 Thermo Scientific, Waltham, MA, USA) for 10 min at RT for the nuclei counterstaining. Then, coverslips were fixed on microscope slides with a PBS‐glycerol solution (3:1; v:v). The images were acquired with a Zeiss microscope through the Zen lite software (Zeiss, Wetzlar, Germany).

A quantitative analysis was performed from Oil Red O staining by measuring absorbance in the same treatment conditions. Cells were seeded on 96‐well plates at a density of 1 × 10^4^ cells/well. At the end of 72 h of treatment, cells were incubated with the working solution (Oil Red O: dH_2_O, 2:3, v:v) for 30 min in the dark. After five washes in distilled H_2_O to remove excess dye, 50 μL of isopropanol was added to each well and left under stirring for 10 min at RT. LDs quantification is obtained by Clariostar Plus Microplate Reader (BMG Labtech, Ortenberg, Germany); the optical density (OD) was measured at 510 nm.

### 2.7 RNA Extraction and RT‐PCR Analysis

Total RNA was extracted using Tri‐Reagent (Sigma‐Aldrich, St. Louis, MO, USA). RNA concentration and purity were detected using the NanoDrop Lite Spectrophotometer (Thermo, Dreieich, Germany). For each sample, 1 μg of total RNA was reverse transcribed using Xpert cDNA Synthesis Supermix (GRiSP Research Solutions, Porto, Portugal) following the manufacturer's instructions. The expression of mAChR subtypes in human astrocytes was evaluated by RT‐PCR. Primers and GoTaq Green 2X Master G2 (Fisher Molecular Biology, Trevose, PA, USA) were added to 100 ng of cDNA.

Below are reported the sequences of the primers used for the RT‐PCR:

*18S*: forward, 5′‐CCAGTAAGTGCGGGTCATAAGC‐3′
  reverse, 5′‐AACGATCCAATCGGTAGTAGCG‐5′
*M1 mAChR*: forward, 5′‐AGAGAGACCCTGCCAACTTT‐3′
  reverse, 5′‐CTCCTGACTTTCCTGCCTAAA‐3′
*M2 mAChR*: forward, 5′‐CCAAGACCCCGTTTCTCCAAG‐3′
  reverse, 5′‐CCTTCTCCTCTCCCCTGAACAC‐3′
*M3 mAChR*: forward, 5′‐CGCTCCAACAGGAGGAAGTA‐3′
  reverse, 5′‐GGAGTTGAGGATGGTGCTGT‐3′
*M4 mAChR*: forward, 5′‐AATGAAGCAAGAGCGTCAAGAA‐3′
  reverse, 5′‐TCATTGGAAGTGTCCTTATCA‐3′
*M5 mAChR*: forward, 5′‐CCTGGCTGATCTCCTTCATC‐3′
  reverse, 5′‐GTCCTTGGTTCGCTTCTCTG‐3′

The expression of SREBP‐1 and SREBP‐2 in GB cells was evaluated by qPCR. qPCR was performed with SYBR Green Mastermix (cat. no. M7822; Promega, Madison, WI, USA) and primers (final concentration 200 nM) were added at the respective wells and analyzed by Thermo Fisher Quantstudio3 (Waltham, MA, USA). Quantification was expressed as 2^−∆∆CT^, where ∆∆CT = ∆CTsample − ∆CTcalibrator. The primers used for qPCR are the following:

*18S*: forward, 5′‐CCAGTAAGTGCGGGTCATAAGC‐3′
  reverse, 5′‐AACGATCCAATCGGTAGTAGCG‐5′
*SREBP1*: forward, 5′‐GGGGACAAGGAATTCTCGGA‐3′
  reverse, 5′‐CTGAACCCTCAGTCACGCT‐3′
*SREBP2*: forward, 5′‐CTCTCCTTTAACCCCCTGAC‐3′ reverse,5′‐CTGACTCGAATGACAGGACA‐3′

### 2.8 Protein Extraction and Western Blot Analysis

Cells were harvested in lysis buffer (Tris‐EDTA 10 mM, 0.5% NP‐40, NaCl 150 mM), containing a protease inhibitor, boiled for 5 min at 90°C. The protein extracts are run on SDS‐polyacrylamide gel (SDS‐PAGE) and transferred to Polyvinylidene Difluoride (PVDF) sheets (Merck Millipore, Darmstadt, Germany). The percentage of SDS‐PAGE used for M2 mAChR detection was 10% and for BNIP3 analysis 15%. Membranes were blocked for 40 min in 5% non‐fat milk powder (Sigma‐Aldrich, St. Louis, MO, USA) in PBS containing 0.1% Tween‐20 (PBS‐Tween), and then incubated overnight at 4°C with one of the following primary antibodies: anti‐β‐Actin (dilution 1:2000, cat. no. MAB‐24008 Immunological Sciences, Rome, Italy), anti‐BNIP3 (dilution 1:800, cat. no. AB‐83988 Immunological Sciences, Rome, Italy), anti‐mAChR M2 (dilution 1:800, cat. no. NB 120‐2805 Novus Biologicals, Centennial, CO USA, RRID: AB_791271), anti‐FAS (dilution 1:1000, cat. no. AB‐84412 Immunological Sciences, Rome, Italy) and anti‐MGAL (dilution 1:1000, cat. no. AB‐84413 Immunological Sciences, Rome, Italy). The blots were washed 3 times with PBS‐Tween then incubated with secondary antibodies conjugated to horseradish‐peroxidase for 1 h. β‐Actin was used as a reference protein. The reaction was revealed by ECL chemiluminescence reagent (cat. no. ECL‐2001; Immunological Science, Rome, Italy). The bands were detected by exposition to Chemidoc (Molecular Imager ChemiDoc XRS + System with Image Lab Software, Biorad, CA, USA) and band intensities were quantified by optical density using ImageJ software Version 1.52a (National Institutes of Health, Bethesda, Maryland, USA).

### 2.9 Cell Viability Assays

Human astrocytes were seeded on poly‐ l‐lysine‐coated 96‐well plates at the density of 5 × 10^3^ cells/well. After 24 h, cells were treated with 100 μM APE, 100 or 25 μM N‐8‐Iper, and also 20 μM Iperoxo (the nonselective, muscarinic super agonist) at different times, ranging from 24 to 72 h. Cell proliferation was evaluated by a colorimetric assay based on 3‐(4,5‐dimethylthiazol‐2‐yl)‐2,5‐diphenyltetrazolium bromide (MTT, Sigma‐Aldrich, St. Louis, MO, USA) metabolization. The MTT assay was performed according to the protocol optimized by Mosmann (Mosmann 1983). MTT was dissolved in PBS at 5 mg/mL. The MTT stock solution (10×) was added and diluted (1×) in each well and then incubated at 37°C for 3 h. Isopropanol (+ 0.04 M HCl + 1% Triton X‐100) was added to all wells and mixed thoroughly to dissolve the dark blue crystals. For each well, the OD at 570 nm was measured by Multiskan FC (Thermo Fisher Scientific, Waltham, MA, USA). The possible toxicity of APE and N‐8‐Iper on human astrocytes was assessed by a viability assay using Erythrosin B staining. Cells were treated with 100 μM APE, 100 μM or 25 μM N‐8‐Iper and 20 μM Iperoxo for 72 h; at the end of treatment, the floating and adherent cells were collected and stained with Erythrosin B (Logos Biosystems, Gyeonggi‐do, South Korea), which stains only dead cells. The percentage of dead cells was analyzed by LUNA‐FX7 Automated Cell Counter (Logos Biosystems, Gyeonggi‐do, South Korea).

### 2.10 Statistical Analysis

Data are representative of three independent experiments and are presented as average ± standard error of the mean (SEM) or standard deviation (SD). Normality was assessed with the D'Agostino–Pearson omnibus *K* ^2^ test, and variance equality was evaluated using Bartlett's test. Statistical analysis was performed by One‐way ANOVA followed by Dunnett multiple comparison post‐test or a Tukey multiple comparison post‐test; when homoscedasticity was not met, Welch ANOVA followed by Dunnett's T3 multiple comparisons test was applied. For non‐parametric datasets, Kruskal–Wallis test with Dunn's post hoc correction was used. Student's *t*‐test was used for pairwise comparisons. Data were considered statistically significant at * *p* < 0.05, ** or # *p* < 0.01 and *** or $ *p* < 0.001. Data analyses were performed with GraphPad Prism 9 (GraphPad Software, La Jolla, CA, USA). Complete statistical informations are provided in Supporting Information (Tables S1–S7).

## 3 Results

### 3.1 ROS Production Induced by M2 mAChR Activation in GB Cells

**FIGURE 1:**
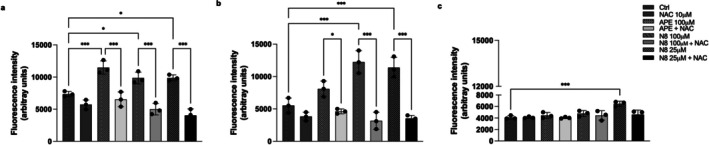
Measurement of ROS levels in (a) G166 cells (b) U251 cell line and (c) human astrocytes after 100 μM APE and 100 μM or 25 μM N‐8‐Iper (N8) treatments. Cells were treated for 12 h in the presence or absence of 10 μM NAC. Data are the average ± SEM of three independent experiments conducted in sextuplicate (the dot points represented the medium value of each of three independent experiments). One‐way ANOVA test followed by the Tukey multiple comparison post‐test was used to statistically compare the different experimental conditions (***p < 0.001, *p < 0.05). Complete statistical information is provided in Table S1.

Our previous results demonstrated that APE and N‐8‐Iper caused cell death in GB cell lines. To clarify the mechanisms downstream M2 mAChR activation, we first analyzed the ability of APE and N‐8‐Iper to induce intracellular ROS production in GSCs (G166 cells) and in GBM stable cell line (U251 cells) (Figure 1). N‐8‐Iper was tested at two concentrations: 100 μM, to compare the effects at the same concentration of APE, and 25 μM, representing the first effective dose in terms of reduction of cell proliferation in both G166 cells (Guerriero et al. 2021) and U251 cell line (Guerriero et al. 2023). The capability of GB cells to produce ROS has been evaluated by DCFH‐DA staining. This experiment was designed from previously reported experiments on the U251 cell line, in which we showed that APE (at concentrations ranging from 25 to 100 μM) induced ROS production after 2 h of treatment (Di Bari et al. 2015). However, when we tested N‐8‐Iper in the same experimental conditions in the U251 cell line and in G166 GSCs, we did not observe significant changes compared with the untreated condition (data not shown). When we extended the treatment time with N‐8‐Iper to 12 h, we detected a significant increase in ROS both in G166 (Figure 1a), in U251 (Figure 1b) and GB7 cells (Figure S1). The increase was counteracted by the action of ROS scavenger NAC (10 μM). Interestingly, at this time point of treatment, the effect of APE was no longer detectable in U251 cell line, indicating that M2 mAChR activation through the orthosteric agonist causes an earlier increase of ROS levels compared to the dualsteric one. We performed the same experiment in human astrocytes. The graph in Figure 1c shows that treatment with 100 μM APE or N‐8‐Iper for 12 h did not produce alteration in ROS production compared to the control; only the treatment with 25 μM N‐8‐Iper caused a faint but significant increase. In any case, the samples treated with ROS scavenger NAC did not show any differences in ROS production in comparison with respective experimental condition.

### 3.2 Analysis of Mitochondrial Activity and Morphology in GB Cells

A persistent increase in ROS can cause multiple effects on mitochondrial components leading to the depolarization of mitochondrial membrane potential (MMP), which can induce mitophagy and/or cell death (Zorov et al. 2014; Zorova et al. 2018). To assess whether the M2 mAChR activation by APE or N‐8‐Iper altered the mitochondrial activity, we tested the MMP after 30 h of treatment with 100 μM APE, 100 μM, or 25 μM N‐8‐Iper. Considered that significant GB cell death was detected from 48 h of M2 agonist treatment (Di Bari et al. 2021), we chose to analyzed the mitochondrial activity and morphology upon 30 h of M2 agonist addition.

**FIGURE 2:**
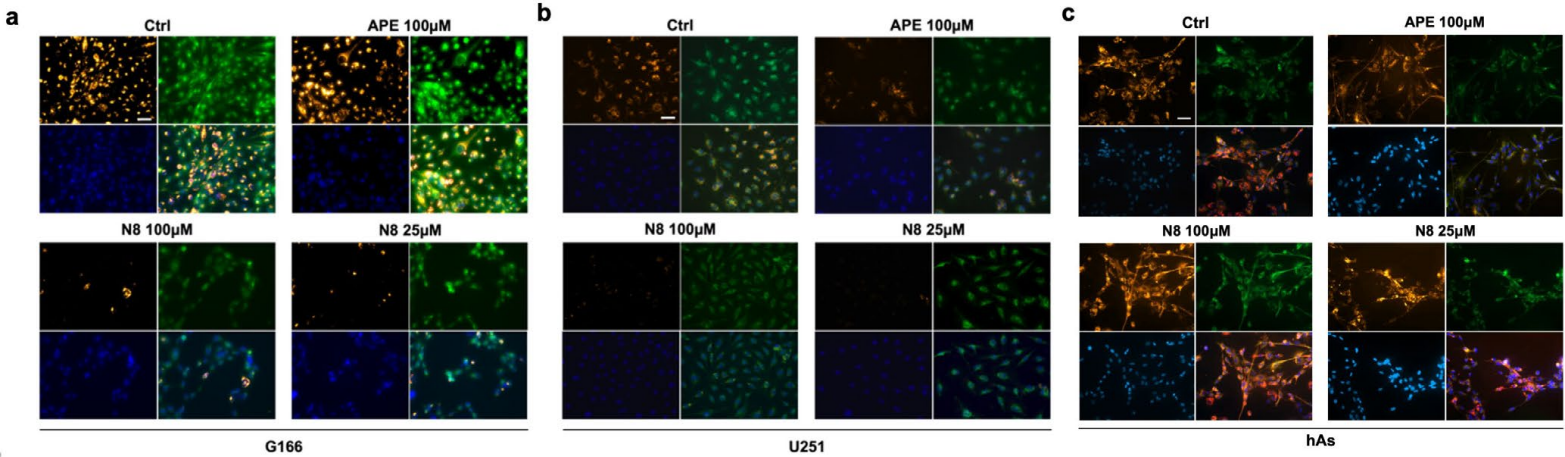
Mitochondria stained with MITO‐ID in (a) G166 cells, (b) U251 cell line and (c) human astrocytes in the control condition and after 30 h of 100 μM APE, and 100 μM or 25 μM N‐8‐Iper (N8) treatments and visualized by a fluorescence microscope. Orange fluorescent aggregates are localized in the mitochondria, while green, fluorescent monomers mainly stain the cytosol. Nuclei were staining by Hoechst 33342. Scale bars = 50 μm.

The MMP was first evaluated using fluorescence measures using the Mito ID detection kit. This MMP assay allows to distinguish between green fluorescent monomers in the cytosol and orange fluorescent aggregates in the mitochondria, a condition indicative of the presence of polarized mitochondria. Figure 2a shows a strong reduction of MMP in G166 cells after 30 h of treatment with the high (100 μM) and low (25 μM) doses of N‐8‐Iper, as orange fluorescence was almost completely lost. Unlike N‐8‐Iper, 100 μM APE treatment did not alter the mitochondrial potential of G166 cells, as both green and orange fluorescence were observed like in the control condition. A similar effect was observed in the U251 cell line (Figure 2b), where MMP was strongly impaired after 30 h of treatment with both APE and N‐8‐Iper (25 μM and 100 μM), as evidenced by both the Mito ID detection kit. Indeed, in Figure 2b, the orange fluorescent signal was clearly reduced after APE treatment, and it almost disappeared in presence of N‐8‐Iper. Except for the control condition, the dye appeared mainly as a green fluorescent monomer throughout the cytosol and no orange fluorescence at the mitochondrial level was detected. Similar results were also observed in another GSC cell line (GB7 cells) (Figure S1).

In human astrocytes, both APE and N‐8‐Iper at both concentrations were not able to alter mitochondrial function, at variance with the result obtained in GB cells. In fact, orange fluorescence was observed under all conditions, proving an active MMP (Figure 2c).

**FIGURE 3:**
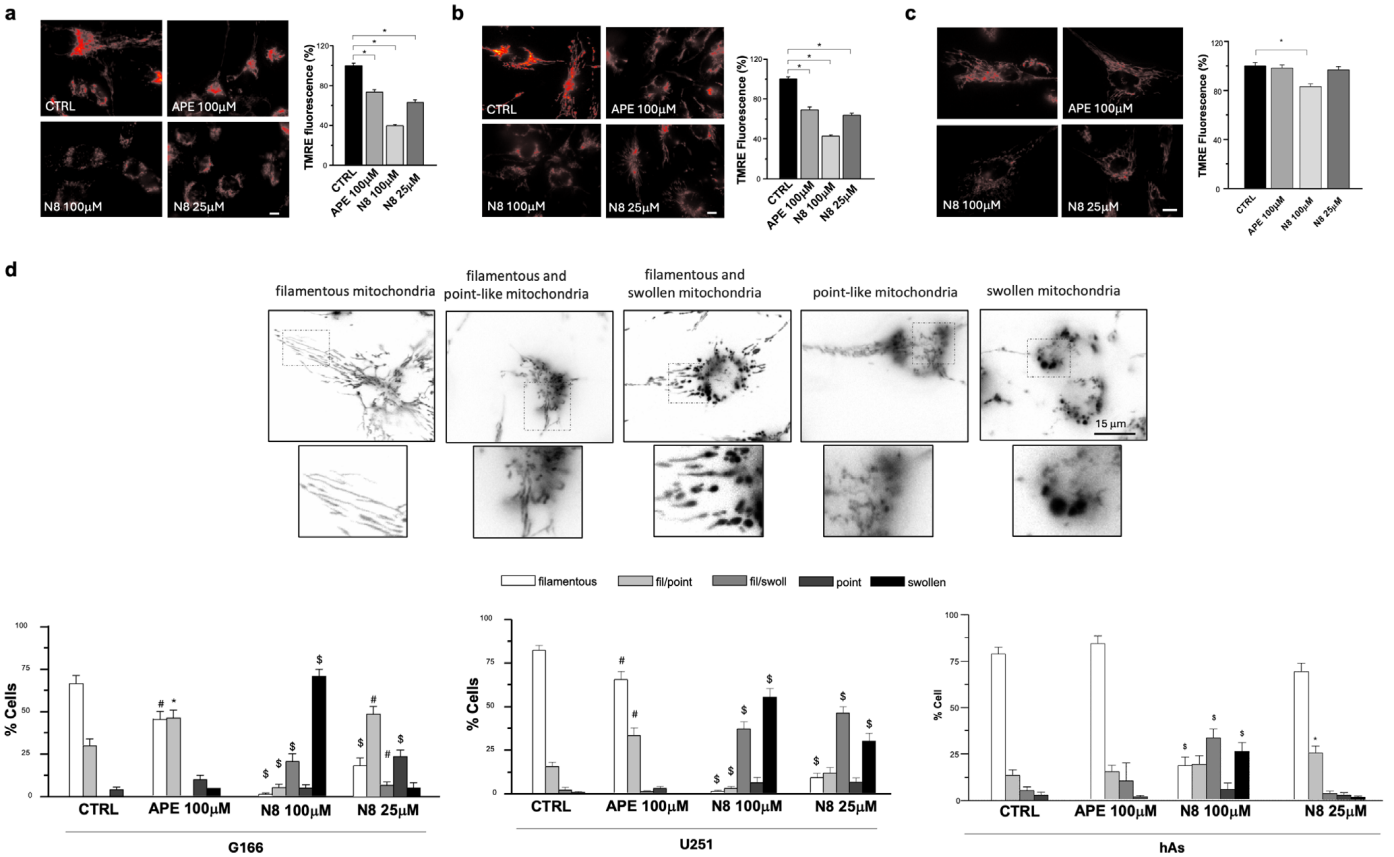
MMP was assessed by the TMRE video‐imaging technique in the control condition and after 30 h of treatment with 100 μM APE, 100 μM N‐8‐Iper (N8), 25 μM N‐8‐Iper (N8) in (a) G166 cells (b) U251 cell line and (c) human astrocytes (hAs). Fluorescence images of TMRE uptake within mitochondria under different experimental conditions are representative. Scale bar = 10 μm. Histograms show the quantification in the percentage of TMRE fluorescence intensity. Data are expressed as the average (± SEM) from 120 mitochondria for each condition; normality was evaluated using the D'Agostino–Pearson test and was not met; therefore, data were analyzed using the non‐parametric Kruskal–Wallis test with Dunn's post hoc correction (treated cells vs. untreated cells, Ctrl. ***p < 0.0001). Complete statistical information is provided in Table S2 (Figure S2) (d) Morphological analysis of mitochondria. Top: Photomicrographs showing TMRE‐loaded mitochondria; inserts showing mitochondrial morphology at higher magnification. Scale bar = 15 μm. Bottom: Distribution of altered mitochondria in G166, U251 and hAs according to mitochondrial morphology under different treatments. Fil, filamentous mitochondria; fil/point, Filamentous and point‐like mitochondria; fil/swoll, filamentous and swollen mitochondria; point, point‐like mitochondria; swoll, swollen mitochondria. Data are mean ± SEM from 129 to 255 cells of three independent experiments. In the graph for human astrocytes, the reported values represent the mean ± SEM from 158 to 180 cells of three independent experiments; normality was evaluated using the D'Agostino–Pearson test and was not met; therefore, data were analyzed using the non‐parametric Kruskal–Wallis test with Dunn's post hoc *p < 0.05, $ *p* < 0.001 calculated within each morphological category (filamentous, filamentous and point‐like, filamentous and swollen, point‐like, swollen mitochondria) versus untreated cultures. Complete statistical information is provided in Tables S2 and S3.

MMP was also measured by the video‐imaging technique using the fluorescent cationic dye TMRE perchlorate (Figure 3). This technique allows mitochondria to be observed at higher magnification, and both MMP and mitochondrial morphology can be analyzed with greater accuracy. Figure 3a shows that in G166 cells treated with both M2 mAChR agonists, the intensity of TMRE‐induced fluorescence decreased when compared with the untreated cells: a reduction of about 27% with 100 μM APE, 37% with 25 μM N‐8‐Iper, and 61% with 100 μM N‐8‐Iper was assessed. These data, therefore, demonstrate that both APE and N‐8‐Iper induce mitochondrial depolarization in G166 cells.

Figure 3b shows a significant decrease in TMRE‐promoted fluorescence following stimulation of M2 mAChR in the U251 cell line, confirming the data from the MITO‐ID assay.

It is known that, in response to cellular and environmental stresses, mitochondria undergo functionally related morphological changes (Picard et al. 2013). When the morphology of mitochondria was inspected with TMRE (Figure 3d), clear differences in shape were noticed in response to the treatment with both M2 mAChR agonists. Figure 3e shows the cell distribution according to the mitochondrial morphology in different conditions. The cell types considered for this analysis are cells with filamentous mitochondria, cells with filamentous and point‐like mitochondria, cells with filamentous and swollen mitochondria, cells with point‐like mitochondria, and cells with swollen mitochondria. In untreated G166 cultures, most cells (about 66.3%) were characterized by mitochondria with a filamentous shape, while in APE‐treated cultures, the majority of cells presented both filamentous and point‐like mitochondria together and cells with filamentous mitochondria (45.2% and 44.7%, respectively). In N‐8‐Iper treated cultures, a significant decrease in the percentage of cells with filamentous mitochondria, coupled with a relevant increase in the percentage of cells with fragmented (point‐like or swollen) mitochondria, was observed with respect to untreated cultures, suggesting an accumulation of dysfunctional mitochondria. In detail, low N‐8‐Iper dose (25 μM) induced mainly an increase in cells with point‐like mitochondria and cells with filamentous and point‐like mitochondria (48.1% and 23.1%, respectively), whereas high doses (100 μM) induced mainly an increase of about 69.8% in cells with swollen mitochondria. In U251 cultures, the effect on the cellular distribution exerted by the orthosteric agonist APE and the dualsteric agonist N‐8‐Iper (25 μM and 100 μM) followed a similar, albeit attenuated, profile to that observed in G166 cells (Figure 3d).

As regards the healthy control cell line, human astrocytes, even when the video‐imaging technique was used, no change was detected in MMP after treatment with both M2 mAChR agonists; only at 100 μM, N‐8‐Iper treatment for 30 h induced a slight, but significant depolarization (16% decrease in TMRE fluorescence vs. untreated sample) (Figure 3c). When the morphology of mitochondria was inspected, we observed that treatment with 100 μM APE and 25 μM N‐8‐Iper did not induce significant differences. Only 100 μM N‐8‐Iper treatment caused a decrease in the percentage of cells showing filamentous mitochondria and an increase in the percentage of cells showing fragmented mitochondria (Figure 3d). These data suggest that 100 μM N‐8‐Iper was the only condition that slightly affected mitochondria in human astrocytes.

### 3.3 Analysis of the Rate of Oxygen Consumption

Changes in mitochondrial morphology and membrane potential suggest mitochondrial dysfunction and mitochondrial damage caused by the M2 agonists. To identify mitochondrial dysfunction, mitochondrial respiration was studied by means of real‐time detection of oxygen consumption rate (OCR) in live cells by the Seahorse XFp analyzer. The Cell Mito Stress Kit was used to identify critical respiratory defects. This test allowed us to collect multiple parameters, including basal respiration, ATP production, and spare respiratory capacity.

**FIGURE 4:**
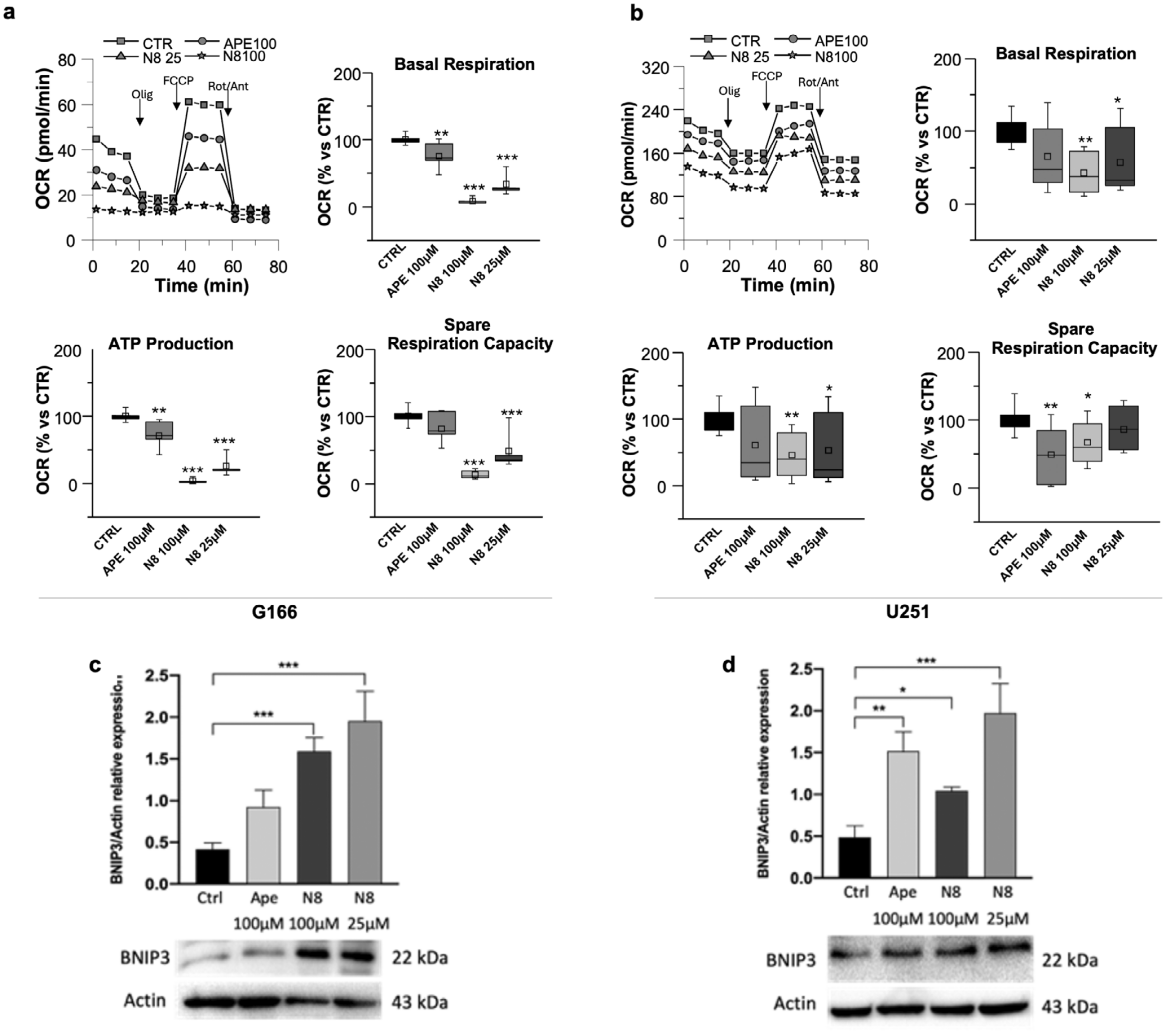
OCR of (a) G166 cells and (b) U251 cell line in control condition and after treatments using a Seahorse XFp analyzer during the consecutive addition of oligomycin (OLIG), carbonyl cyanide 4‐(trifluoromethoxy)phenylhydrazone (FCCP), and rotenone (ROT) + antimycin A (ANT). Data are the average (± SEM) of three independent experiments. In the box plots, the boxes represent the interquartile range (25th–75th percentiles), the central line indicates the median, the square represents the mean, and the whiskers show the full range (minimum to maximum). Normality was assessed with the D'Agostino–Pearson omnibus *K* ^2^ test, and variance equality was evaluated using the Brown–Forsythe test. Because data were normally distributed but showed unequal variances, group differences were analyzed using Welch's ANOVA followed by Dunnett's T3 multiple comparisons test (treated cells vs. untreated cells, * *p* < 0.05, **p < 0.01, ***p < 0.001). Western blot analysis of BNIP3 expression after 30 h of treatment with 100 μM APE, 100 μM or 25 μM N‐8‐Iper (N8) in (c) G166 cells and in (d) U251 cell line. Actin was used as an internal reference protein. The graphs show the densitometric analysis of the bands of western blot analysis for BNIP3 normalized with the bands of Actin. Data are the average (±SEM) of three independent experiments. ANOVA test was used, followed by Dunnett's post‐test (treated cells vs. untreated cells, Ctrl. ***p < 0.001, **p < 0.01; *p < 0.05). Complete statistical information is provided in Tables S4 and S4 bis.

To assess whether the M2 mAChR activation by APE or N‐8‐Iper altered mitochondrial respiration, we measured the OCR after 30 h of treatment with 100 μM APE, 100 μM or 25 μM N‐8‐Iper (Figure 4a,b). In G166 cells (Figure 4a), after treatment with N‐8‐Iper, a strong reduction was observed in basal mitochondrial respiration, mitochondrial ATP production and spare respiratory capacity, suggesting that the agonist induces not only a decrease in basal conditions in the cell energy demand, with a consequent decrease in ATP production, but also a decrease in the ability to respond to an increase in energy demand. It is worth noting that the effects of N‐8‐Iper on mitochondrial respiration were dose‐dependent. Treatment with 100 μM APE, instead, induced a milder effect on basal mitochondrial respiration and on mitochondrial ATP production, while, unlike N‐8‐Iper, the orthosteric agonist did not appear to alter the spare respiratory capacity. As shown in Figure 4b, in U251 cells the stimulation of M2 mAChR by N‐8‐Iper induced a significant reduction in basal mitochondrial respiration and in mitochondrial ATP production; moreover, only the high dose induced a significant effect on spare respiratory capacity, even though at low dose a decreasing trend was observed. Instead, the orthosteric agonist APE, unlike N‐8‐Iper, did not induce any significant effect on the observed parameters, except for the spare respiratory capacity. These data suggest that, in G166 and U251 cultures, both agonists alter mitochondrial respiration, although with different intensities.

High oxidative stress and mitochondrial damage can lead to mitophagy, a selective autophagy of mitochondria (Wang et al. 2020). By Western blot analysis, we assessed the expression of BNIP3, one of the factors involved in the FUNDC1/BNIP3/NIX pathway, which targets the mitochondria for mitophagy. As shown in Figure 4c,d, the protein level of BNIP3 was low in untreated cells. In G166 cells (Figure 4c), BNIP3 expression was upregulated after treatment with N‐8‐Iper, where mitochondrial damage was observed. On the other hand, in the U251 cell line (Figure 4d), the increase in BNIP3 protein level was observed in the presence of both M2 agonists.

### 3.4 Analysis of Lipid Accumulation

Increased ROS production, mitochondrial damage, and oxidative stress promote the formation of LDs, neutral lipid storage organelles, mostly composed of triacylglycerols (TAGs) and sterol esters (Liu et al. 2015). Oil red O staining detected LDs within the cells under examination. Based on the timing of the cellular response to stress induced by both APE and N‐8‐Iper, we treated cells for 72 h to assess lipid accumulation.

**FIGURE 5:**
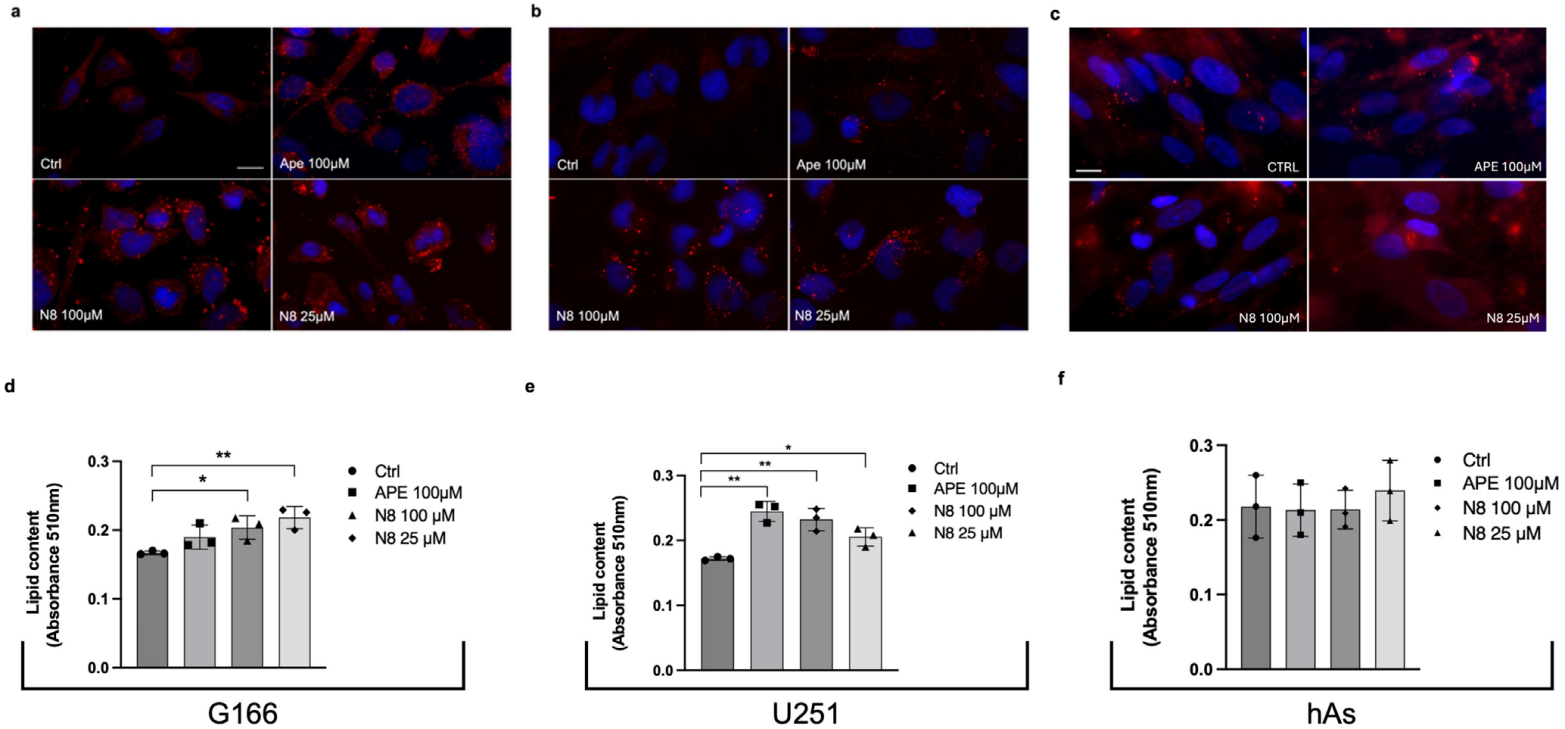
Oil red O staining in (a) G166 cells, (b) U251 cell line and (c) human astrocytes in control condition and after 72 h of treatment with 100 μM APE, 100 μM or 25 μM N‐8‐Iper (N8). Images were acquired using a Zeiss fluorescence microscope. Scale bar = 10 μm. Quantitative analysis of LDs content in (d) G166 cells, (e) U251 cell line and (f) human astrocytes in control condition and after 72 h of treatment with 100 μM APE, 100 μM or 25 μM N‐8‐Iper (N8). Quantification was obtained using a multimodal microplate reader (CLARIOstar Plus, BMG Labtech). Data are the average ± SEM of three independent experiments conducted in sextuplicate in the graphs are reported the medium values of three independent experiments. ANOVA test was used, followed by Dunnett's post‐test (treated cells vs. untreated cells, Ctrl. **p < 0.01; *p < 0.05). Complete statistical information is provided in Table S5.

The Oil red O staining showed an increase in LDs accumulation both in G166 cells (Figure 5a) and in the U251 cell line (Figure 5b) after APE and N‐8‐Iper treatments. The LDs content has been quantified through Oil red O staining, measuring the dye absorba within the cells. This analysis put in evidence a significant increase in fluorescence after treatment with N‐8‐Iper in both G166 cells (Figure 5d) and the U251 cell line (Figure 5e), and with APE only in the U251 cell line.

The analysis of lipid accumulation by Oil red O staining was also conducted on human astrocytes. Analysis of both imaging (Figure 5c) and fluorescence readout (Figure 5f) does not show an altered accumulation of LDs within the cells after activation of M2 mAChR, both with APE and N‐8‐Iper.

**FIGURE 6:**
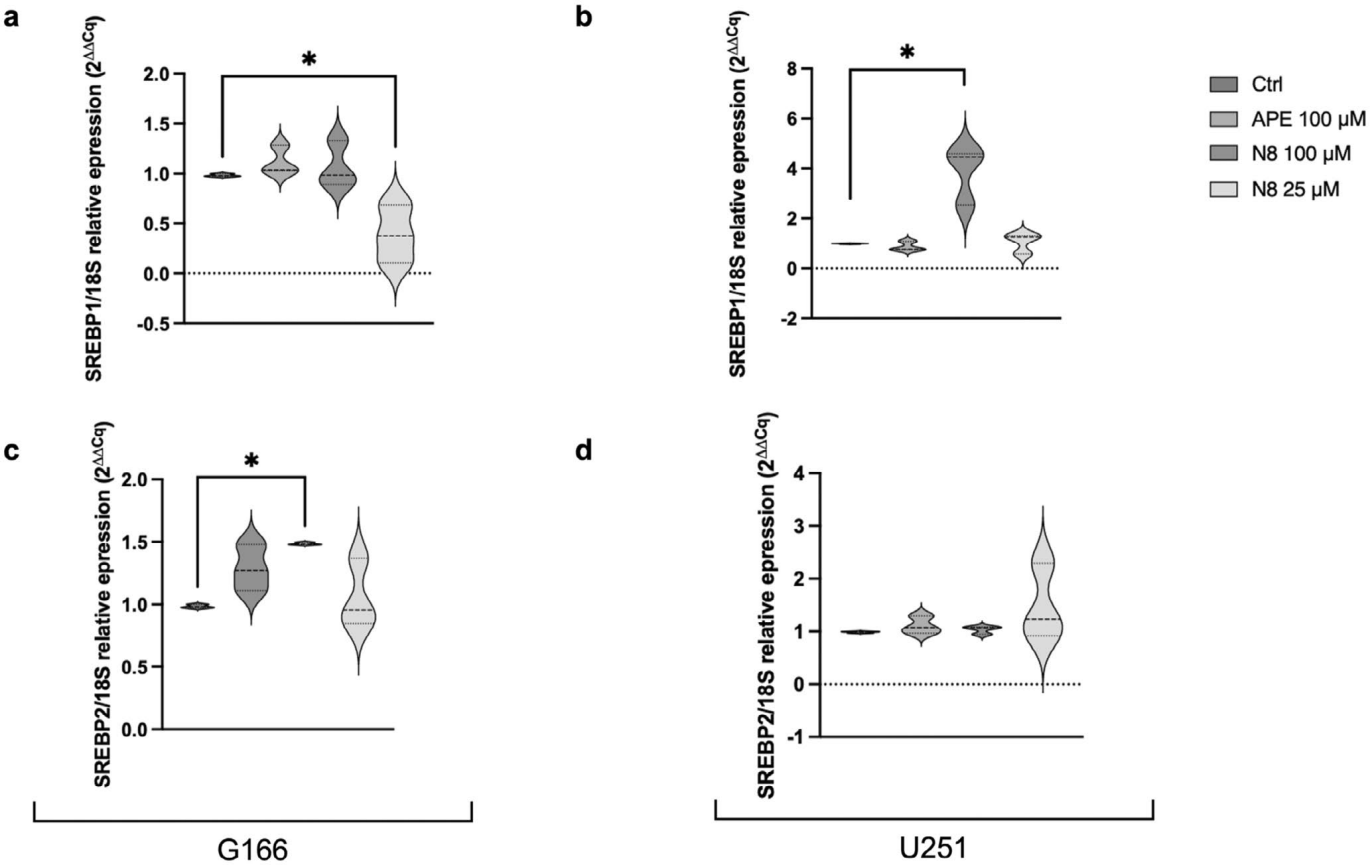
qPCR analysis for SREBP1 (a, b) and SREBP2 (c, d) in G166 cells (a, c) and U251 cell line (b, d). Data are the average ± SEM of three independent experiments conducted in triplicate; the ANOVA test was used, followed by Dunnett's post‐test (treated cells vs. untreated cells, Ctrl. * *p* < 0.05).

To ascertain whether the lipid accumulation depended on altered lipid metabolism, we analyzed the mRNA expression of Sterol regulatory element‐binding transcription factors 1 (SREBP1) and 2 (SREBP2), that are involved in the regulation of the synthesis of fatty acids and cholesterol (He et al. 2024). The qPCR in Figure 6 shows that the regulation of fatty acid and cholesterol biosynthesis is in general unchanged, even if 100 μM of N8‐Iper caused an upregulation of SREBP2 in GB166 and of SREBP1 in U251 cells. SREBP1 appeared significantly decreased in GB166 after 25 μM of N8‐Iper.

**FIGURE 7:**
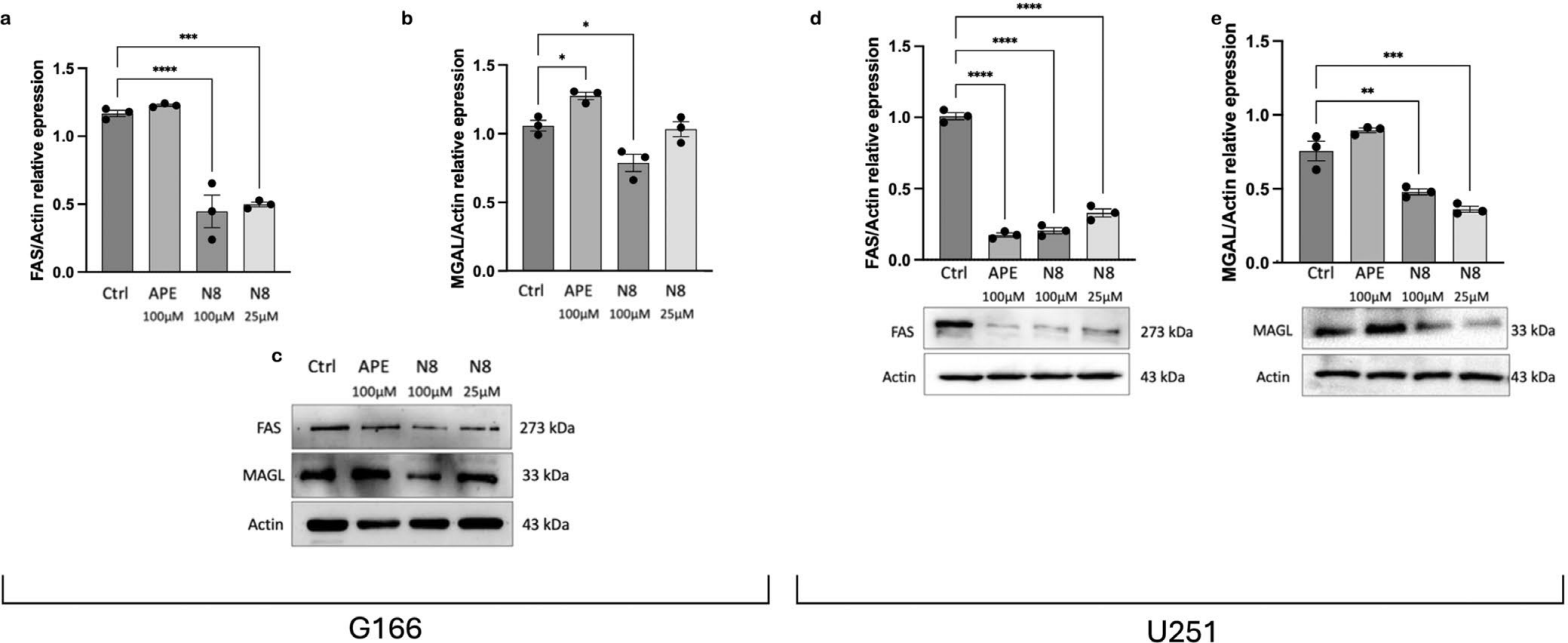
Western blot analysis of FAS and MAGL expression after 72 h of treatment with 100 μM APE, 100 μM N‐8‐Iper (N8), 25 μM N8 in G166 cells (a, b, c) and in U251 cell line (d, e), respectively. Actin was used as internal reference protein. The graphs show the densitometric analysis of the bands of western blot analysis for FAS or MAGL normalized with the bands of the Actin. The data are the average (±SEM) of three independent experiments. ANOVA test was used followed by Dunnett's post test (treated cells vs. untreated cells, Ctrl. ***p < 0.001; **p < 0.01; *p < 0.05). Complete statistical information is provided in Table S5.

In order to better understand the possible modification of lipid metabolism after M2 mAChR activation, we evaluated, by western blot analysis, the protein expression of two enzymes involved in this process, the multi enzymatic complex fatty acid synthase (FAS) and monoacylglycerol lipase (MGAL). As shown in the Figure 7c, G166 cells treated with 100 μM APE for 72 h did not show any change in FAS protein expression compared with control, according to SRBP1 expression (Figure 6a). Conversely, the treatment with 100 μM N‐8‐Iper or 25 μM N‐8‐Iper caused a significant reduction in FAS expression both in G166 and U251 cells (Figure 7a,c). Regarding MAGL, in G166 cells, as observed in the Figure 7b, treatment with APE 100 μM for 72 h caused an increase in MAGL protein expression, whereas downregulation of MAGL was observed in cells treated with 100 μM N‐8‐Iper. No alteration was observed after treatment with N‐8‐Iper 25 μM compared with the control condition in G166 (Figure 7b). In the U251 cell line, the downregulation of MAGL protein expression was also observed after N‐8‐Iper treatment at high and low doses (Figure 7d).

### 3.5 Analysis of Cell Proliferation and Survival in Human Astrocytes

**FIGURE 8:**
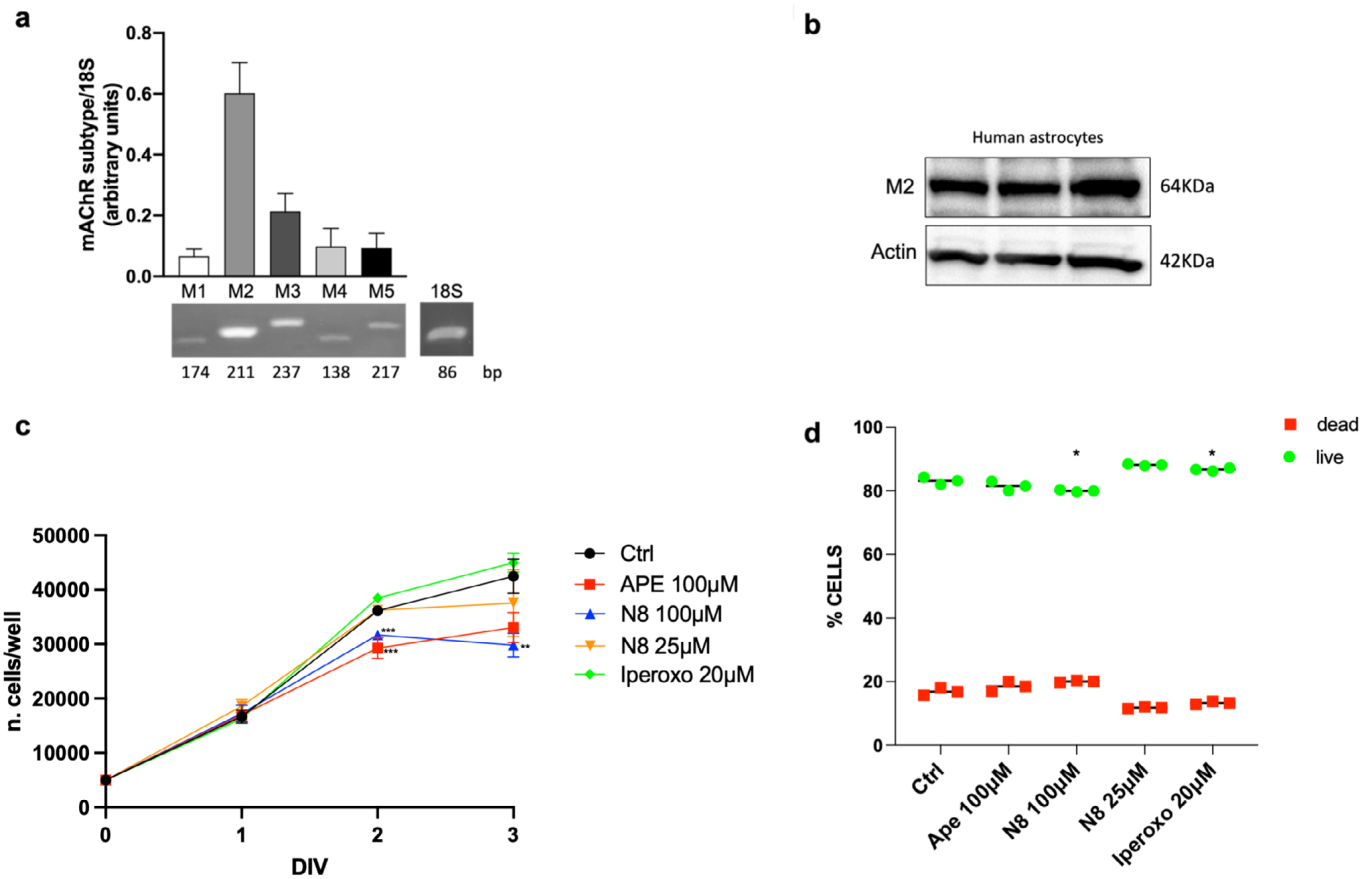
(a) RT‐PCR analysis of five mAChR subtypes transcripts (M1, M2, M3, M4, M5) in human astrocytes. 18 s was used as the housekeeping gene. The gel figures are representative of three independent experiments. Densitometric analysis was obtained from the average ± SEM of three independent experiments. (b) M2 mAChR protein expression by Western blot analysis in three different protein extracts obtained from human astrocyte cultures. β‐Actin was used as the internal reference protein. (c) Effect of APE (100 μM), N‐8‐Iper (100 μM or 25 μM) and Iperoxo (20 μM) on human astrocyte cell growth at different times of treatment (ranging from 1 to 3 DIV = days in vitro). Data represent the mean (± SEM) of three different experiments performed in quadruplicate. ANOVA test was used followed by Dunnett's post‐test (treated cells vs. untreated cells, Ctrl. ***p < 0.001; **p < 0.01). (d) Cell viability was assessed by cell counting. Human astrocytes were plated in triplicate, treated with 100 μM APE, and 100 μM or 25 μM N‐8‐Iper or 20 μM Iperoxo. After 72 h of treatment, cells were counted by LUNA‐FX7 using Erythrosin B dye. The graph shows the percentage of dead cells in the different experimental conditions (Three independent experiments performed in quadruplicate. The dot points are represented by the medium values of each of three independent experiments). Student's *t*‐test was used to statistically compare the different experimental conditions (*p < 0.05). Complete statistical information is provided in Tables S6 and S7.

The experiments performed in GB cells were repeated in human astrocytes, which represent the healthy cell population compared to the tumor population under investigation. We focused on the effects induced by low‐dose N‐8‐Iper, and characterized M2 mAChRs on human astrocytes both in terms of expression, by RT‐PCR and Western blot analysis, and of function, in relation to their ability to modulate cell proliferation and viability, by MTT and Erythrosin B staining viability assay, respectively (Figure 8).

The expression of the five mAChR subtypes (M1‐M5) was analyzed by means of RT‐PCR; as observed in Figure 8a, human astrocytes exhibit all mAChR subtypes, with the M2 mAChR being the most highly expressed at the transcript level.

Expression of the M2 mAChR protein, assessed by western blot analysis, confirmed the presence of the M2 subtype in human astrocytes (Figure 8b).

Next, we evaluated whether the treatment of normal human astrocytes with the two M2 agonists showed the same cytotoxic effects observed in GB cells. For this purpose, an MTT assay to analyze cell proliferation (Figure 8c) and a viability assay by Erythrosin B staining (Figure 8d) were performed. The high dose (100 μM) of both APE and N‐8‐Iper was chosen to compare their effects. The first effective dose of N‐8‐Iper (25 μM) on the tumor cell lines and treatment with the nonselective super agonist (20 μM Iperoxo) were also evaluated.

The MTT assay showed no significant alterations in cell proliferation after 24 h of treatment (1 DIV) with M2 agonists at all concentrations tested. After 48 h of treatment (2 DIV), only the high dose of both M2 agonists caused a significant decrease in cell growth. As shown in Figure 8c, only 100 μM N‐8‐Iper was able to induce a significant decrease in cell growth after 72 h of treatment (3 DIV = day in vitro). The low doses of N‐8‐Iper did not impair cell growth at any of the experimental times analyzed.

After 72 h of treatment, under the same experimental conditions as those of the MTT assay, cell viability was assessed by staining with Erythrosin B dye with a LUNA‐FX7 Automated Cell Counter. As can be realized from the graph in Figure 8d, no significant decrease in cell viability was detected between the M2 agonist‐treated cells and the control sample, except for 100 μM N‐8‐Iper, which caused a slight but statistically significant increase of the percentage of dead cells at 72 h of treatment.

## 4 Discussion

Our previous data showed that the selective M2 mAChR activation, mediated by the orthosteric agonist APE, induces cytotoxic effects, decreasing cell proliferation and causing cell cycle arrest in both GB cell lines (U87 and U251) and GSCs (GB7 and GB8). However, APE acted at high (50–100 μM) doses only (Ferretti et al. 2013; Alessandrini et al. 2015). Albeit these results identified the M2 mAChR as a promising target, a more powerful and selective muscarinic ligand is needed in view of putative therapeutic applications. To this aim, in recent years, our group has investigated the effects of the synthetic M2 mAChR dualsteric agonist N‐8‐Iper (Guerriero et al. 2021; Cristofaro et al. 2018). Already from the first analysis, N‐8‐Iper was shown to act similarly but at significantly lower doses than APE. Indeed, GB7 cells showed a significant reduction in cell proliferation upon 72 h treatment with N‐8‐Iper at the dose of 3 μM (Cristofaro et al. 2018), and, even in the most resistant GSCs, G166 cells, N‐8‐Iper reduced cell proliferation at lower doses (25 μM) than APE (Guerriero et al. 2021).

In this work, we evaluated the modulation of some intracellular mechanisms following M2 mAChR activation that may help deepen the investigation of the already discussed cytotoxic effects in GB cells (Guerriero et al. 2023), focusing on the possible different effects produced by orthosteric agonist APE and dualsteric N‐8‐Iper in order to better explain the higher efficacy of the latter agonist. Building on the ability of APE to induce oxidative stress in the U251 cell line after 2 h of treatment (Di Bari et al. 2015), we found that this agonist failed to act in GSCs at the same experimental time. In fact, only after 12 h of 100 μM APE treatment was a significant increase in intracellular ROS release evident in G166. Similarly, N‐8‐Iper was able to significantly increase intracellular ROS in both cell lines at the same treatment time. These results confirmed the ability of two M2 agonists to increase oxidative stress in GB cells, albeit the effect of the dualsteric agonist was evident at a longer time of treatment (Figure 1).

Mitochondria are the most important intracellular source of ROS and produce the superoxide anion as a byproduct of mitochondrial respiration (Afonso et al. 2007). Unbuffered superoxide production by mitochondria is responsible for pathological oxidation of respiratory chain complexes and Krebs cycle enzymes, causing further production of ROS, decreased respiratory efficiency, ATP production, and depolarization of inner membrane potential. This latter was shown to concur with the triggering of mitophagy by causing the translocation into damaged mitochondria of proteins involved in mitophagy (Twig and Shirihai 2011).

Albeit N‐8‐Iper seems to be more efficient, we noted a difference in the ability of APE and N‐8 to induce ROS (2 h with APE, 12 h with N‐8‐Iper). For this reason, the analysis of mitochondrial activity appears relevant to understand how the cytotoxic effects were induced by APE or N‐8‐Iper.

By using several different techniques, we here tried to offer a comprehensive description of the effects on mitochondria that could help to distinguish the different therapeutic potential of the two M2 agonists. However, the disparity among the approaches did not always help designing a unifying picture. When measured with the TMRE‐fluorescence technique, both agonists affected MMP, although with slightly different potency. On the contrary, morphological examination highlighted a stronger effect of N‐8‐Iper even at the low concentration tested. Together with the accumulation of point‐like mitochondria, probably due to fission activity, N‐8‐Iper caused the appearance of a large amount of more detrimental swollen mitochondria, most probably due to permeability transition pore opening and loss of ionic homeostasis (Javadov et al. 2018). The more pronounced toxicity of N‐8‐Iper was also evident when analyzing the parameters obtained by Seahorse experiments, especially in G166 cells: basal respiration, ATP production, and spare respiratory capacity. The agonist induces not only a strong decrease in basal conditions in the energy demand of the cell with a consequent decrease in ATP production but also a significant decrease in the ability to respond to an increase in energy demand.

The ROS release analysis agreed with the results related to MMP, respiration and mitochondrial morphology (Figures 2, 3, 4), albeit the effects of N‐8‐Iper (at high and low doses) were more pronounced than the orthosteric agonist APE. Damaged mitochondria increase the expression of BNIP3, a protein involved in regulating mitophagy. According to previous data on mitochondrial function alterations, the dualsteric agonist N‐8‐Iper was found to increase BNIP3 expression, suggesting mitophagy activation in both cell lines; conversely, APE had the same effect only in the U251 cells. These results are not surprising considering that, as demonstrated by our previous data, APE induced stronger cytotoxic effects in the p53 mutated GB cell lines (i.e., U251 cells and GB8 GSCs) (Di Bari et al. 2015, 2021). Conversely, cancer stem cells are endowed with relevant DNA repair properties, and, in particular, G166 cells are characterized by high resistance to chemotherapy treatments (Guerriero et al. 2021; Conti et al. 2012; Pollard et al. 2009). By virtue of this property, it should not be surprising that, although sensitive to the effect of N‐8‐Iper, G166 cells respond to a higher concentration of N‐8‐Iper (25 μM) than that found in GB7 cells (3 μM) (Cristofaro et al. 2018). The link existing between M2 mAChR activation and the drastic alteration of mitochondrial activity will be the subject of future in‐depth studies, but from these data, mitochondria seem to play a central role in explaining the different effects of the two muscarinic ligands.

As a consequence of the alteration in mitochondrial activity and oxidative stress induction following N‐8‐Iper treatment, we found a significant increase in LDs accumulation with N‐8‐Iper in both G166 cells and U251 cell line (Figure 5). Accumulation of LDs may be a consequence of oxidative stress, mitochondrial damage and the altered respiration induced by the dualsteric agonist that prompted the GB cells to make use of the lipids as an alternative metabolic resource, as typically described in tumor cells (Wu et al. 2020).

*De novo* lipogenesis is required for tumor growth; for these reasons, inhibitors of enzymes involved in the synthesis of the *de novo* fatty acid in cancer cells provide a novel therapeutic approach causing cell cytotoxicity and cell death (Hathaichanok et al. 2014). Several studies have highlighted the association between FAS expression and tumor activities such as invasion, proliferation, and metastasis (Mozolewska et al. 2020). MAGL has also been shown to be highly expressed in human cancer cells, promoting the survival of cancer cells and tumor growth (Nomura et al. 2010).

Blocking FAS and MAGL activity could therefore have antitumor effects. Increasing lipogenesis as well as the upregulation of lipolytic activity is associated with increased tumor aggressiveness. This process contributes to cancer progression through several mechanisms that utilize the stored fatty acids (Hathaichanok et al. 2014).

Notably, APE treatment significantly increased the amount of LDs compared with untreated cells only in the U251 cell line, confirming its preferential effects on p53 mutated cells. The lack of LDs accumulation in APE‐treated G166 cells is in accordance with the less severe mitochondrial damage, which would be indicative of a lesser efficacy of the orthosteric agonist APE than the dualsteric agonist N‐8‐Iper to induce stress and cytotoxic effects. However, the analysis of SREBP1 and SREBP2 expression, transcription factors capable of stimulating the synthesis of both FA and cholesterol, respectively, indicates that APE has no significant effect on the expression of these factors neither in U251 nor in G166. Instead, N8 100 μM seems to positively modulate SRBP1 in U251 and SRBP2 in G166. While a decrease in SRBP1 is observed in G166 only with a low dose of N8. Analyzing these data in comparison with the expression of the FAS and MGAL enzymes, it is possible to observe that APE induces significant activation of the MGAL enzyme, but has no effect on FA synthesis, consistent with the lack of SRBP1 modulation. In U251 cells, however, APE determines a reduction in the FAS enzyme and no change in MGAL expression. Conversely, high‐dose N8 significantly reduced the FAS and MGAL enzymes in both U251 and G166. Only the 25 μM concentration appeared to have no effect on MGAL. The decrease in FAS expression is at least partially consistent with the decrease in SREBP1 observed in G166 after low‐dose N8 treatment. Our results demonstrate that in particular N‐8‐Iper downregulate the enzymes involved in lipogenesis and in lipolysis, blocking the upstream and downstream the process. This can explain the increase in LDs in N‐8‐Iper‐treated cells. The results obtained suggest that the increased oxidative stress, mitochondrial damage and the altered respiration induced by M2 agonists prompt the GBM cells to use the lipids as metabolic resources. However, lipid accumulation and blockade of both lipid synthesis and degradation lead us to believe that stimulation of M2 mAChR causes lipid accumulation whose failure to degrade could impair the ability of tumor cells, particularly GSCs, to counteract M2 agonist‐induced cytotoxic stress. Instead APE stimulation did not cause a strong lipid accumulation in G166 cells that could be explained by unaltered lipogenesis and increased lipid degradation. In accordance with these results, N‐8‐Iper, at variance with APE, triggers autophagy and apoptosis in GSCs and stable cell lines (Guerriero et al. 2023). This finding suggests that the cytotoxic damage accumulated by tumor cells and lipid accumulation, are not recovered and drives them to death. Albeit APE induces oxidative stress, it causes less severe mitochondrial damage compared to N‐8‐Iper, suggesting that the respiration, and the mitochondrial and metabolic functions are not completely compromised in the presence of APE. Therefore APE‐induced apoptosis in GB cells, may be dependent on other mechanisms that may be p53‐dependent (Alessandrini et al. 2015). However, the orthosteric agonist acts only at high concentrations and is unable to produce effects of comparable intensity in cancer stem cells. Conversely, N‐8‐Iper is by far more efficacious, and triggers significant cytotoxic and genotoxic effects (Cristofaro et al. 2018) both in cancer stem cells and in stable cell lines at lower concentrations than APE. This may due, at least in part, to the additional allosteric interaction of the ligand that could promote “a stimulus bias” causing a recruitment and/or potentiation of different transduction pathways. Remarkably, neither APE nor N‐8‐Iper engender cytotoxic effects in normal human astrocytes. Indeed, both M2 agonists caused only a faint slowdown of astrocyte proliferation but no significant increase in cell death (Figure 7) or mitochondrial alteration (Figures 2 and 3), or LDs accumulation was observed (Figure 5). Only 100 μM N‐8‐Iper induced a slight modification of mitochondrial function and morphology, while 25 μM N‐8‐Iper, that is, the concentration of potential interest for the treatment of GSCs, did not produce any significant alteration, suggesting the absence of toxic effects on normal human astrocytes with low doses of this dualsteric muscarinic ligand.

In conclusion, albeit further analysis performed in tumor organoids and in preclinical animal models are necessary, our data confirm that M2 mAChRs may represent an alternative and promising therapeutic tool for GB and highlight the efficacy profile of the dualsteric agonist N‐8‐Iper in counteracting the growth and survival of cancer stem cells without affecting the viability of healthy astrocytes.

## Author Contributions

**Claudia Guerriero:** data curation, investigation, writing – original draft. **Chiara De Nuccio:** data curation, investigation, methodology, writing – review. **Maria Petrone:** investigation. **Teresa Rinaldi:** data curation, methodology. **Angela Cirigliano:** methodology. **Sergio Visentin:** methodology, writing – review and editing. **Antonietta Bernardo:** methodology, writing – review and editing. **Luciano Conti:** methodology, writing – review and editing. **Carlo Matera:** methodology. **Marco De Amici:** methodology, writing – review and editing. **Clelia Dallanoce:** methodology. **Ada Maria Tata:** conceptualization, resources, supervision, writing – review and editing.

## Consent

Informed consent was achieved for all subjects, and the experiments were approved by the local ethics committee.

## Conflicts of Interest

The authors declare no conflicts of interest.

## Supporting information


**Figure S1:** jnc70454‐sup‐0001‐Supinfo01.pdf.

## Data Availability

The datasets used and/or analyzed during the current study are available at the corresponding author on reasonable request.
