# Supplementary material for: Dualsteric Agonist for M2 Muscarinic Receptor Causes Oxidative Stress and Mitochondrial Alteration in Human Glioblastoma Cancer Stem Cells
Source: J Neurochem. 2026 May 16;170:e70454. doi: 10.1111/jnc.70454 (PMC13179530; doi:10.1111/jnc.70454)

JNC-2025-0361 Supplementary materials

Dualsteric agonist for M2 muscarinic receptor causes oxidative stress and mitochondrial alteration in human glioblastoma cancer stem cells

Claudia Guerriero; Chiara De Nuccio, Maria Petrone, Teresa Rinaldi, Angela Cirigliano, Sergio Visentin, Antonietta Bernardo, Luciano Conti, Carlo Matera, Marco De Amici, Clelia Dallanoce, and Ada Maria Tata*

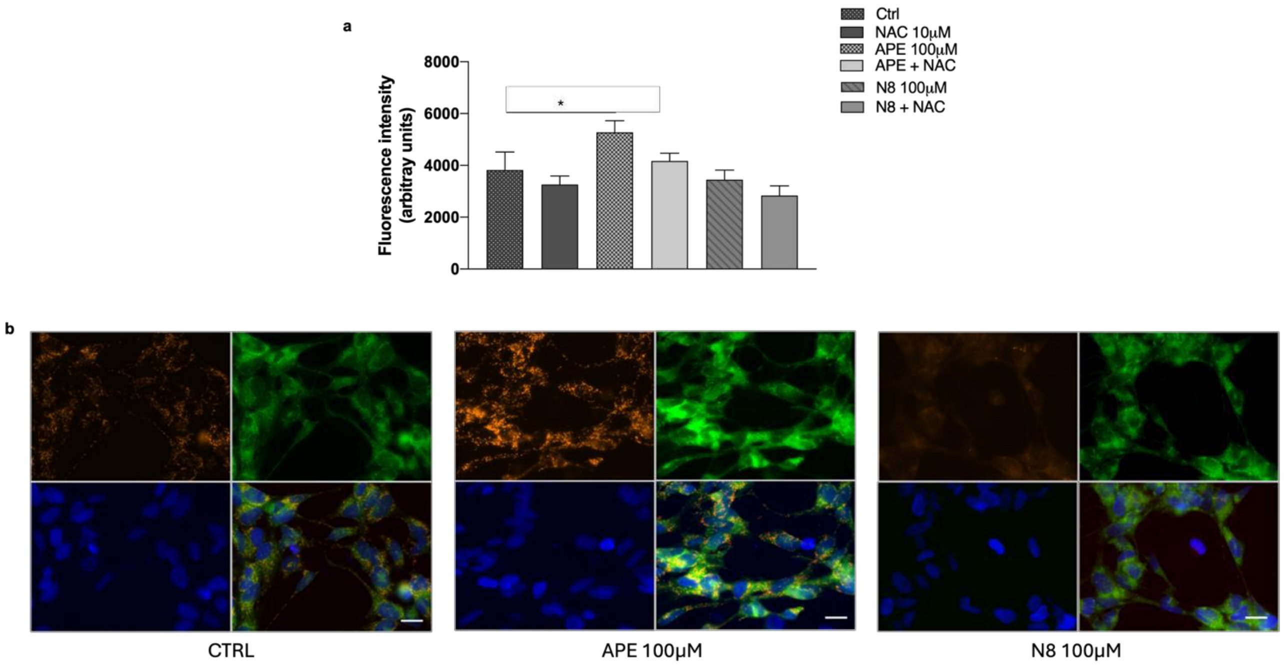

FIGURE S1: **(a)** Measurement of ROS levels in GB7 cells **(**after 100 μM APE and 100 μM N-8-Iper (N8) treatments. Cells were treated for 12 h in the presence or absence of 10 μM NAC. Data are the average ± SEM of three independent experiments conducted in sextuplicate. One-way ANOVA test followed by the Tukey multiple comparison post-test was used to statistically compare the different experimental conditions (* $p < 0.05$). **(b)** Mitochondria stained with MITO-ID® in GB7 cells in the control condition and after 30 h of 100μM APE and 100 μM N-8-Iper (N8) treatments and visualized by a fluorescence microscope. Orange fluorescent aggregates are localized in the mitochondria, while green, fluorescent monomers mainly stain the cytosol. Nuclei were staining by Hoechst 33342. Scale bars = 50 μm.

- Statistical information-

cells	Parameter	ANOVA test		Tukey's multiple comparison post-test - P value						
		F(df, df)	P value	ctrl vs NAC	ctrl vs APE 100µM	APE 100µM vs APE + NAC	ctrl vs N8 100µM	N8 100µM vs. N8 100µM+ NAC	ctrl vs N8 25µM	N8 25µM vs. N8 25µM + NAC
G166	ROS	28.29 (7, 16)	<0.0001	0.3403	0.0005	<0.0001	0.0431	<0.0001	0.0420	<0.0001
U251		28.91 (7, 16)	<0.0001	0,6274	0.1917	0.0341	<0.0001	<0.0001	0.0003	<0.0001
hAs		3.227 (7, 125)	0.0035	0.9996	0.9775	0.7921	0.4855	0.7921	0.0353	0.4862

Table S1 Measurement of ROS levels in G166 cells, U251 cell line and human astrocytes after 100 µM APE and 100 µM or 25 µM N-8-Iper (N8) treatments. Data are the average ± SEM of three independent experiments conducted in sextuplicate. For the statistical analysis were used One-way ANOVA test followed by the Tukey multiple comparison post-test to statistically compare the different experimental conditions. The table reports the statistic details, degrees of freedom, and p value. Post-hoc analysis was performed using Tukey's multiple comparisons test; the table shows the test type, the group-to-group comparison, and the corresponding actual p values.

Cells	Parameter	Kruskal–Wallis test		Dunn's multiple comparisons test - P value		
		H(df)	P value	ctr vs APE	ctr vs N8 100µM	ctr vs N8 25µM
G166	MMP	209.3 (3)	<0.0001	<0.0001	<0.0001	<0.0001
U251		245.5 (3)	<0.0001	<0.0001	<0.0001	<0.0001
hAs		23.47 (3)	<0.0001	>0.9999	<0.0001	>0.9999

Table S2. Statistical analysis of MMP levels in G166, U251, and hAs cells following treatment with APE, N8 100 µM, and N8 25 µM (Figure 3a, 3b and 3c) Normality was assessed using the D'Agostino–Pearson omnibus K^2 test. As the data did not follow a normal distribution, group differences were evaluated using the Kruskal–Wallis nonparametric test. The table reports the H statistic, degrees of freedom, and P value. Post-hoc analysis was performed using Dunn's multiple comparisons test; the table shows the test type, the group-to-group contrasts evaluated, and the corresponding actual P values.

Cells	Mitochondrial morphology	Kruskal–Wallis test		Dunn's multiple comparisons test - P value		
		H(df)	P value	ctr vs APE	ctr vs N8 100µM	ctr vs N8 25µM
G166	filamentous	48.63 (3)	<0.0001	0.1059	<0.0001	<0.0001
	filamentous and point-like	34.83 (3)	<0.0001	0.0489	0.0054	0.0386
	filamentous and swollen	44.01 (3)	<0.0001	>0.9999	<0.0001	0.0458
	point-like	17.24 (3)	0.0006	0.4639	>0.9999	0.0004
	swollen	62.79 (3)	<0.0001	>0.9999	<0.0001	>0.9999
U251	filamentous	83.75 (3)	<0.0001	0.3831	<0.0001	<0.0001
	filamentous and point-like	32.88 (3)	<0.0001	0.0499	0.0027	0.5061
	filamentous and swollen	80.55 (3)	<0.0001	>0.9999	<0.0001	<0.0001
	point-like	3.109 (3)	0.3751	0,7001	0.9344	0.2633
	swollen	70.59 (3)	<0.0001	>0.9999	<0.0001	<0.0001
hAs	filamentous	53.71 (3)	<0.0001	>0.9999	<0.0001	0.5138
	filamentous and point-like	6.495 (3)	0.0898	>0.9999	>0.9999	0.0668
	filamentous and swollen	53.58 (3)	<0.0001	0.6194	<0.0001	>0.9999
	point-like	0.8537 (3)	0.8366	>0.9999	>0.9999	>0.9999
	swollen	57.22 (3)	<0.0001	>0.9999	<0.0001	>0.9999

Table S3. Statistical analysis of mitochondrial morphology distributions in G166, U251, and hAs cells following treatment with APE, N8 100 µM, and N8 25 µM. (Figure 3d) Normality was assessed using the D'Agostino–Pearson omnibus K^2 test. As the data did not follow a normal distribution, group differences were evaluated using the Kruskal–Wallis nonparametric test. The table reports the H statistic, degrees of freedom, and P value. Post-hoc analysis was performed using Dunn's multiple comparisons test; the table shows the test type, the group-to-group contrasts evaluated, and the corresponding actual P values.

Cells	Parameters	Welch's ANOVA test		Dunnett's T3 multiple comparisons test - P value		
		W (DFn, DFd)	P value	ctr vs APE	ctr vs N8 100µM	ctr vs N8 25µM
G166	Basal Respiration	209.4 (3.000, 12.07)	<0.0001	0.028	<0.0001	<0.0001
	ATP Production	247,4 (3.000, 11.66)	<0.0001	0.009	<0.0001	<0.0001
	Spare Respiration Capacity	75.95 (3.000, 11.86)	<0.0001	0.254	<0.0001	0.0015
U251	Basal Respiration	7.073 (3.000, 12.81)	0.0048	0.160	0.016	0.030
	ATP Production	7.638 (3.000, 12.45)	0.0037	0.210	0.015	0.024
	Spare Respiration Capacity	6.031 (3.000, 13.03)	0.0083	0.0144	0.038	0.583

Table S4. Statistical analysis of Bbasal respiration, ATP production, and spare respiratory capacity levels in G166 and U251 cells following treatment with APE, N8 100 µM, and N8 25 µM (Figure 4a, 4b). Normality was assessed with the D'Agostino–Pearson omnibus K^2 test, and variance equality was evaluated using the Brown–Forsythe test. Because data were normally distributed but showed unequal variances, group differences were analyzed using Welch's ANOVA, followed by Dunnett's T3 multiple comparisons test. The table reports the W statistic, degrees of freedom (DFn, DFd), and the actual P value for each Welch's ANOVA comparison. Post-hoc results include the test type, all pairwise group comparisons, and their corresponding actual P values.

cells	Parameter	ANOVA test		Dunnett's multiple comparison post-test - P value		
		F(df, df)	P value	ctrl vs APE 100μM	ctrl vs N8 100μM	ctrl vs N8 25μM
G166	BNIP3 protein expression	24.37 (3, 8)	0.0002	0.0697	0.0008	0.0001
U251		20.93 (3, 8)	0.0004	0,.0016	0.0406	0.0002

Table S4 bis. Statistical analysis related to Western blot of BNIP3 expression after 30 h of treatment with 100 μM APE, 100 μM or 25 μM N-8-Iper (N8) in G166 cells and in U251 cell line. Data are the average (±SEM) of three independent experiments.

For the statistical analysis were used One-way ANOVA test followed by the Dunnett's multiple comparison post-test to statistically compare the different experimental conditions. The table reports the statistic details, degrees of freedom, and p value. The table shows the test type, the group-to-group comparison, and the corresponding p values.

cells	Parameter	ANOVA test		Dunnett's multiple comparison post-test - P value		
		F(df, df)	P value	ctrl vs APE 100μM	ctrl vs N8 100μM	ctrl vs N8 25μM
G166	FAS protein expression	46.33 (2, 8)	<0.0001	0.8312	<0.0001	0.0002
	MAGL protein expression	17.36 (3, 8)	0.0007	0.0313	0.0100	0.9632
U251	FAS protein expression	287.1 (3, 8)	<0.0001	<0.0001	<0.0001	<0.0001
	MAGL protein expression	44.23 (2, 8)	<0.0001	0.0678	0.0019	0.002

Table S5. Statistical analysis related to Western blot of FAS and MAGL expression after 72 h of treatment with 100 μM APE, 100 μM N-8-Iper (N8), 25 μM N8 in G166 cells and in U251 cell line. The data are the average (±SEM) of three independent experiments.

For the statistical analysis were used One-way ANOVA test followed by the Dunnett's multiple comparison post-test to statistically compare the different experimental conditions. The table reports the statistic details, degrees of freedom, and p value. The table shows the test type, the group-to-group comparison, and the corresponding p values.

Cells	Time of treatment	Parameter	ANOVA test		Dunnett's multiple comparison post-test - P value			
			F(df, df)	P value	ctrl vs APE 100μM	ctrl vs N8 100μM	ctrl vs N8 25μM	ctrl vs Iperoxo 20μM
hAs	24h	Cells growth (MTT test)	1.845 (4, 10)	0.1969	0.9932	0.9150	0.1811	0.9758
	48h		38.35 (4, 10)	<0.0001	<0.0001	0.0015	0.9999	0.0645
	72h		9.470 (4, 10)	0.0020	0.0278	0.0047	0.3172	0.8020

Table S6. Statistical analysis relative to MTT assay in Astrocytes. Data represent the mean (± SEM) of three different experiments performed in quadruplicate. ANOVA test was used followed by Dunnett's multiple comparison post-test to statistically compare the different experimental conditions. The table reports the statistic details, degrees of freedom, and p value. The table shows the test type, the group-to-group comparison, and the corresponding p values.

cells	Parameter	Comparisons	Unpaired t test	
			**t, df**	**P value**
hAs	Viability	**ctrl vs APE 100μM**	1.512, 4	0.2051
		**ctrl vs N8 100μM**	4.602, 4	0.0100
		**ctrl vs N8 25μM**	7.361, 4	0.0018
		**ctrl vs Iperoxo 20μM**	4.918, 4	0.0079

Table S7. Statistical analysis of cell viability in human astrocytes. Student's t-test was used to statistically compare the different experimental conditions. The table reports the statistic details, degrees of freedom, and p value. The table shows the test type, the group-to-group comparison, and the corresponding p values.

Western blot (Original Membranes)

BNIP3 expression

G166

BNIP3

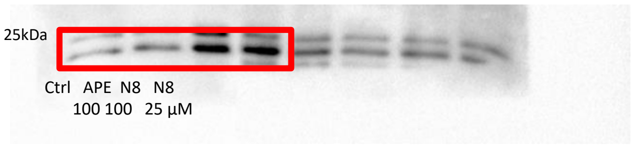

ACTIN

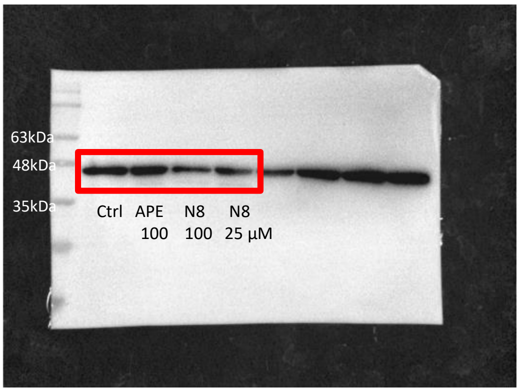

U251

BNIP3

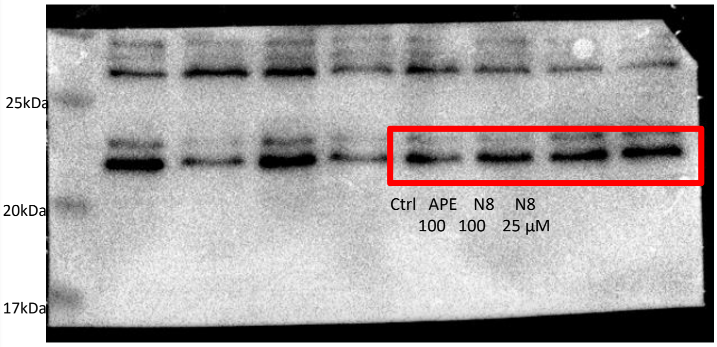

ACTIN

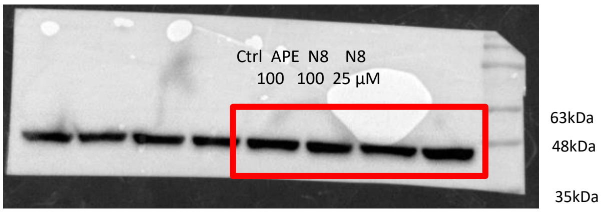

G166
WESTERN BLOT

FAS and MGAL expression

ACTIN

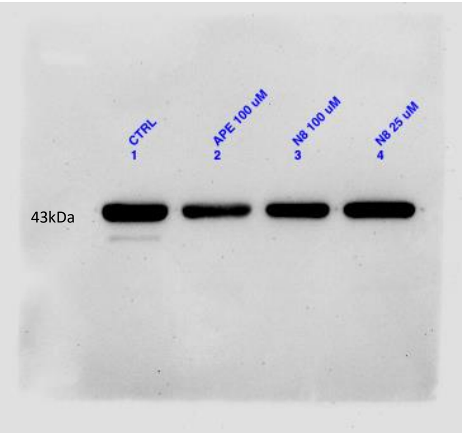

FAS

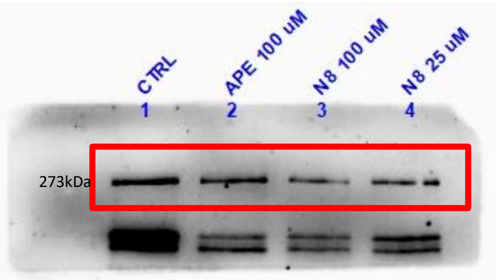

MAGL

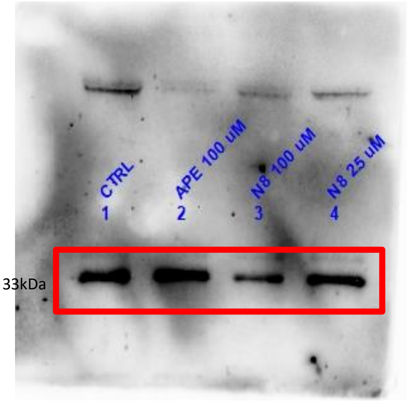

U251
WESTERN BLOT

FAS

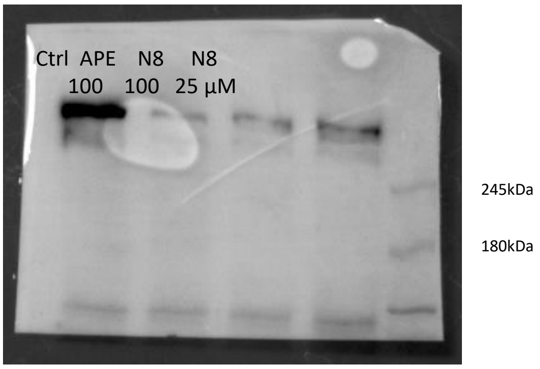

ACTIN

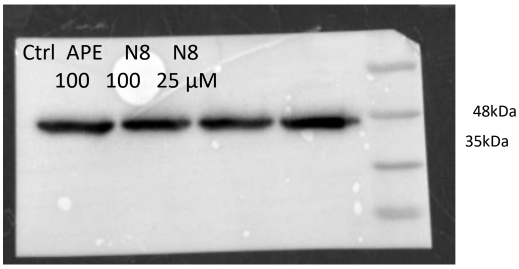

MGAL

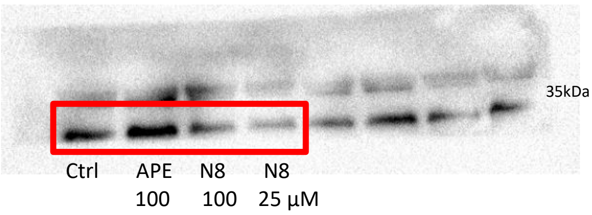

ACTIN

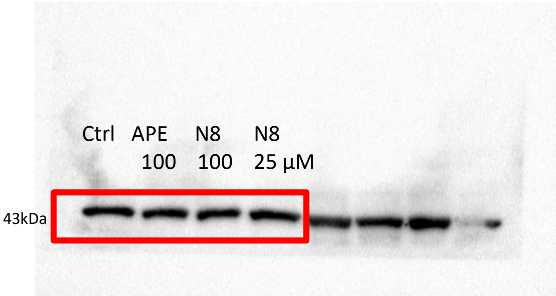

Human astrocytes

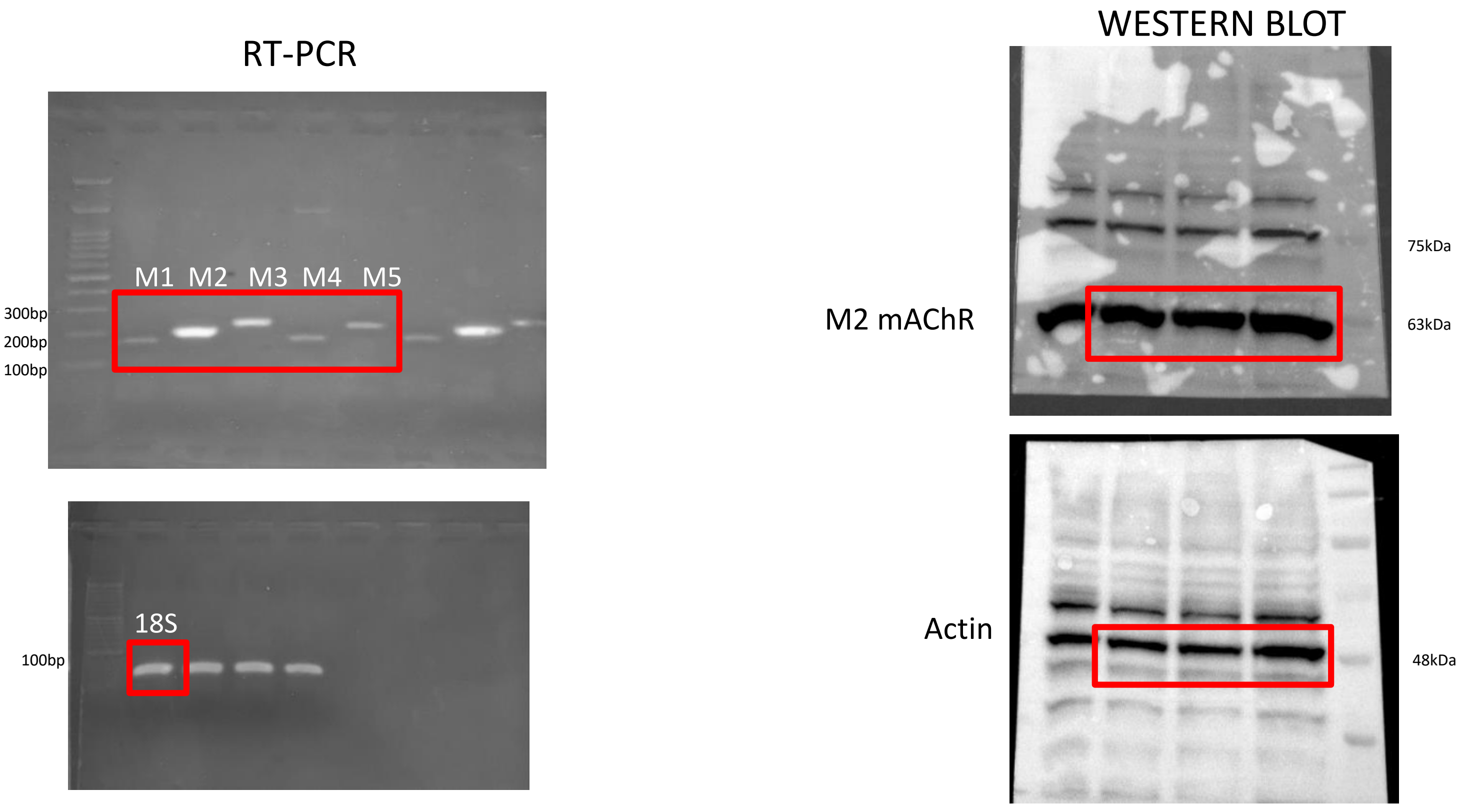

Supplement: Supplementary file 1 — Figure S1: jnc70454‐sup‐0001‐Supinfo01.pdf. [file JNC-170-e70454-s001.pdf]
